# Predictive sampling effort and species-area relationship models for estimating richness in fragmented landscapes

**DOI:** 10.1371/journal.pone.0226529

**Published:** 2019-12-31

**Authors:** Noé U. de la Sancha, Sarah A. Boyle

**Affiliations:** 1 Department of Biological Sciences, Chicago State University, Chicago, Illinois, United States of America; 2 Integrative Research Center, The Field Museum of Natural History, Chicago, Illinois, United States of America; 3 Department of Biology, Rhodes College, Memphis, Tennessee, United States of America; University of Waikato, NEW ZEALAND

## Abstract

Loss of habitat, specifically deforestation, is a major driver of biodiversity loss. Species-area relationship (SAR) models traditionally have been used for estimating species richness, species loss as a function of habitat loss, and extrapolation of richness for given areas. Sampling-species relationships (SSRs) are interrelated yet separate drivers for species richness estimates. Traditionally, however, SAR and SSR models have been used independently and not incorporated into a single approach. We developed and compared predictive models that incorporate sampling effort species-area relationships (SESARS) along the entire Atlantic Forest of South America, and then applied the best-fit model to estimate richness in forest remnants of Interior Atlantic Forest of eastern Paraguay. This framework was applied to non-volant small mammal assemblages that reflect different tolerances to forest loss and fragmentation. In order to account for differences in functionality we estimated small mammal richness of 1) the entire non-volant small mammal assemblage, including introduced species; 2) the native species forest assemblage; and 3) the forest-specialist assemblage, with the latter two assemblages being subsets of the entire assemblage. Finally, we geospatially modeled species richness for each of the three assemblages throughout eastern Paraguay to identify remnants with high species richness. We found that multiple regression power-law interaction-term models that only included area and the interactions of area and sampling as predictors, worked best for predicting species richness for the entire assemblage and the native species forest assemblage, while several traditional SAR models (logistic, power, exponential, and ratio) best described forest-specialist richness. Species richness was significantly different between assemblages. We identified obvious remnants with high species richness in eastern Paraguay, and these remnants often were geographically isolated. We also found relatively high predicted species richness (in relation to the entire range of predicted richness values) in several geographically-isolated, medium-size forest remnants that likely have not been considered as possible priority areas for conservation. These findings highlight the importance of using an empirical dataset, created using sources representing diverse sampling efforts, to develop robust predictive models. This approach is particularly important in geographic locations where field sampling is limited yet the geographic area is experiencing rapid and dramatic land cover changes. When combined, area and sampling are powerful modeling predictors for questions of biogeography, ecology, and conservation, especially when addressing habitat loss and fragmentation.

## Introduction

We are currently in the sixth mass extinction on our planet, the Anthropocene [1]. The Earth’s surface is covered by more than 4 billion ha of forest habitats that account for approximately 31% of the total land area of the planet [2]. Approximately 3.3 million ha of net forest loss occurred from 2010–2015 [2], resulting in the fragmentation of key habitats on the planet. The primary driver for mammal extinction is the loss and fragmentation of habitat [3], and this potentially includes large numbers of poorly known or species yet to be described [4,5]. Thus, how do we account for species that we don’t know exist? This issue is critical in places like Paraguay, where the species assemblages are relatively poorly understood [6]. The Atlantic Forest of South America has experienced extreme levels of deforestation [7]. The second-largest moist forest system in South America after the Amazon [8], the Atlantic Forest extends from northeastern Brazil along the coastline to southern Brazil, and inland into eastern Paraguay and northern Argentina. The Atlantic Forest is considered one of the major “hotspots” for biodiversity in the world [9]. Approximately 12% and 20% of the original Atlantic Forest remains in Brazil and Paraguay, respectively [10,11]. Major changes to the Atlantic Forest from anthropogenic activities did not begin until about the 1940s in Paraguay [12]. The Atlantic Forest in Paraguay was reduced by 30% in 20 years (1970 to 1990) and by 2000 it was reduced to only one fourth of its original extent, mostly due to soybean cultivation [11]. Thus, a logical subsequent question is what is the effect of this deforestation on regional biodiversity?

There are many potential predictors of species richness in fragmented landscapes [13–16], modeling is limited by local or regional understanding of these dynamics and, in most cases, by local datasets. While it is ideal to sample locally for all possible predictors, what do we do when those data do not exist, which is often the situation in many geographical areas around the world? And, more urgently, what can be done in the face of rapid and constant habitat change, as what has happened in the Atlantic Forest of Paraguay [17]? Two well-established and valuable predictors for species richness have included area and sampling [18]. However, there is still debate on whether these are interrelated or completely independent relations [14,18]. In geographic areas that have not been sampled widely, and that are still poorly understood in regard to species composition, finding models that will help prioritize areas for conservation is a valuable and urgent task.

The species-area relationship (SAR) often shows a pattern of increased species richness with increased area, and has been one of the most consistent patterns observed in ecology [19–37] and particularly true for mammals [38]. SAR models have improved our understanding of biodiversity at biogeographical and ecological scales alike [27,28,31,32,37,39,40]. Furthermore, SAR models have been valuable in their application to management and conservation [19,21,23,25,27,28,30,41–48]. Specifically, SAR models can be useful for richness estimates and/or estimates of extinction resulting from habitat fragmentation or habitat loss [32,39,49,50].

However, the use of SAR models is not without controversy and some have found SAR models as unfit for estimating extinction rates after habitat loss [41–48]. For example, some have found that SAR models overestimate real species loss in cases of small-to-moderate habitat loss, while others have found this overestimation occurs regardless of habitat loss size [41,49,50]. Some researchers have concluded that SAR models overestimate extinction by estimating encounter with the first individual of a species [41], versus the removal of the last individual of that species, thus extinction [39,41].

At least two power-law based functions have been proposed for using an endemic species-area relationship (EAR) model instead of a SAR model [22,23,25,27,35,41,49–54]. Additionally, one could use a function that measures the remaining species-area relationship (RAR) [39]. While each of these estimates have their own merits, it is important to note that the power-law, also known as the power function, is not the only nor necessarily the best for SAR models [22,23,25,27,35,51–54].

At least 27 functions have been described to model SARs, most of which are non-linear models [22,27,28,31,32,35,37,40]. However, the general pattern of these relationships has fueled considerable debate about what is the best mathematical function to use and what shape best describes SARs [20,23,24,26,27,29,31,33–37]. The power function [24,27,28,31,32,37,40,55], or some derivative thereof, has gained wide acceptance and popularity as the best function to apply to study SARs, mostly by convention and convenience rather than for biological reasons [19,21,23,25,27,28,30,32,35,37,56]. Many studies have used power function-based SAR models to estimate extinction rates by comparing the proportion of extinctions after habitat loss of a given area to the number of species present in a larger area [32,39,40,54]. However, recent works have shown that the power function is not always “the best fit model” [23,57]. The power function lacks an asymptote, thus producing exceedingly high species estimates for larger areas [54]. Despite preferences in functions, SAR models continue to be the most frequently used approach to predict biodiversity loss in systems that have experienced habitat fragmentation [39,4–48,53,56]. There remains an opportunity to test various models that optimize SARs [23,25,30,49,50,52–54], as the power function and other SAR algorithms may oversimplify or significantly change the fundamental understanding of the diversity patterns in question [23,32,41].

Beyond area, sampling effort is another major factor influencing biodiversity estimates [41,58]. Often, studies focusing on biogeographical or macroecological scales consist of conglomerations of smaller local studies [41,49,50,53,58–64]. Rarely are these smaller local studies based on equal sampling efforts due to differences in study design and duration. However, there is a positive relationship between species richness estimates and sampling effort, where greater sampling efforts typically result in higher richness, also known as the species-sampling effort relationship (SSER) [39,58].

Related and interwoven, as summarized by Azovsky [58], the SSER and SAR vary in that a SSER accounts for richness in a local sampled area (i.e. a grid or trapline) while a SAR is concerned with species heterogeneity increase over the area of habitat or region of focus (i.e. a forest remnant or a bioregion). Variation in SSER models can confound estimates of species richness and thus the nature of the SAR [22,23,25,27,35,51–54,58]. While there are examples of multivariate species estimators [35], rarely, if ever, have SAR models incorporated sampling variation into one model [65]. Combining area and sampling efforts can result in powerful modeling predictors for SARs or EARs for questions of biogeography, ecology, and conservation, as such models allow for the simultaneous manipulation of two important variables for predicting species richness. If sampling was not an improvement in modeling species richness, then one would expect traditional species area models to outperform models that incorporate sampling and area to predict species richness.

While there are considerable empirical data on species richness for the Atlantic Forest as a whole from northeastern Brazil to eastern Paraguay [59,66,67], information about which species are found in Paraguayan forest remnants is lacking in comparison. Our main objective was to multivariate predictive models that would allow us to incorporate sampling and area for the Atlantic Forest, then apply those models to forest remnants in Paraguay, in order to identify regional remnants with high species richness. Second, given that different species have different functionality, our second objective was to model three species assemblages that account for different functions. Our approach allows us to go beyond a species-area model to include a sampling-area-species plane.

The aim of our study was to develop predictive models that incorporate species richness, area, and sampling effort (Sampling effort Species-Area relationship models; SESARS), and use these models to address four interrelated ecological questions that have profound implications for biodiversity conservation: 1) Are species predictive models that include sampling effort improvements over traditional species-area models? 2) What are the best-fit models that include both sampling and area for predicting species richness in the Atlantic Forest of South America? 3) How do different non-volant small mammal assemblages, with different sensitivity to habitat type, respond to deforestation? 4) As a case study, where are the remnants with high non-volant small mammal species richness for the highly fragmented Atlantic Forest of eastern Paraguay?

## Materials and methods

### Workflow overview

**Fig 1:**
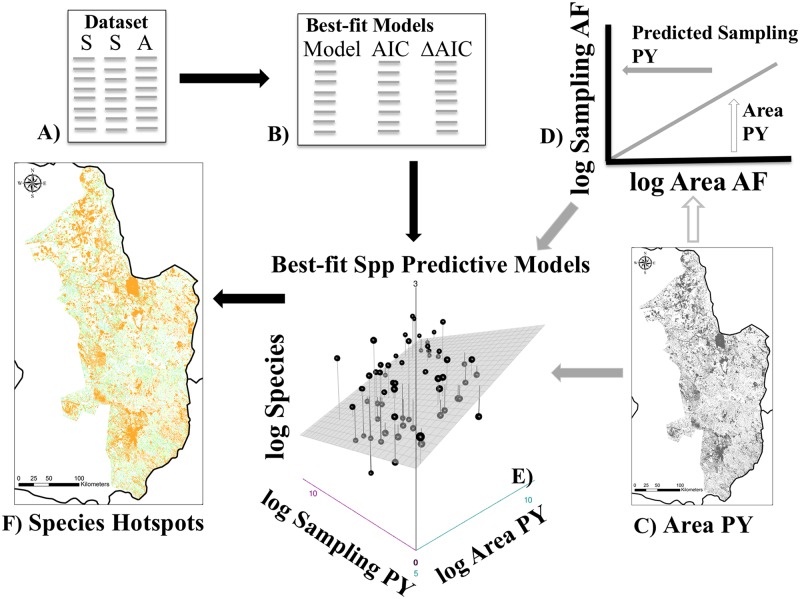
Flowchart of our workflow. A) We began with a dataset of 68 forest remnants through the Atlantic Forest (AF) from 20 published studies (see text for details), where area, species, and sampling efforts were all included for each assemblage and site. B) We tested 14 sampling-species-area (SESARS) models and traditional species-area relationship functions and compared the models via Akaike Information Criterion (AIC) to find the best-fit model. C) We calculated the size of every AF remnant of eastern Paraguay ≤ 0.50 ha from 2014 forest cover data (Hansen et al. 2013). D) Meanwhile we used a log-transformed linear model from the original empirical data for the entire AF to estimate the appropriate sampling efforts that would correspond to the areas estimated from our shapefile of eastern Paraguay. E) Using the corresponding best-fit predictive model, we estimated species richness as a function of area for eastern Paraguay, and proportional sampling effort (when appropriate), from our 2014 forest cover data for eastern Paraguay. F) Finally, we georeferenced estimated species richness for the AF remnants to find remnants with high species richness in eastern Paraguay.

Our workflow (Fig 1) began with the building of predictive models for the entire Atlantic Forest. These models incorporated 20 studies of non-volant small mammals from 68 forest remnants from northeastern Brazil to eastern Paraguay, where area, species richness, and sampling efforts were all included in each study (S1 Table). We used 8 traditional species-area models (see Traditional species-area (SAR) functions; Table 1), 28 linear log and semi-log sampling effort and species-area relationships (SESARS) models (Table 2) and 7 non-linear generalized additive models (see Sampling Effort and Species-Area Relationships (SESARS) models; Table 3), for a total of 43 possible predictive models. We compared all of the multivariate and SAR models among themselves to find the best-fit models, for three separate assemblages of species with varying tolerance to forest loss and fragmentation (see section on Case study dataset). The best-fit models per assemblage were then implemented in a case study of the forest remnants of eastern Paraguay to demonstrate the application of our approach. We used forest cover data from 2014 [68] to generate a georeferenced dataset of the forest remnants of eastern Paraguay. This approach allowed us to predict species richness for all of the forest remnants ≥ 0.50 ha in eastern Paraguay. Finally, we visualized estimated species richness for eastern Paraguay to identify remnants with high species richness for each of the three assemblages (Fig 1).

**Table 1:** Eight traditional species-area relationships used comparative analyses.

Traditional SAR	SAR ID	Formula	Number of Parameters	Shape	Asymptotic nature
Power	Power	SR = cA^z^	2	Convex	No
Exponential	Expo	SR = c+zlog(A)	2	Convex	No
Negative exponential	Negexpo	SR = d(1-exp(zA))	2	Convex	Yes
Monod	Monod	SR = d/(1+cA^-1^)	2	Convex	Yes
Rational function	Ratio	SR = (c+zA)/(1dA)	3	Convex	Yes
Logistic	Logist	SR = d/(1exp(-zAf))	3	Sigmoid	Yes
Lomolino	Lomolino	SR = d/1(z^log(f/A)^)	3	Sigmoid	Yes
Cumulative Weibull	Weibull	SR = d(1-exp(-zA^f^))	3	Sigmoid	Yes

**Table 2:** The sampling effort and species-area relationship (SESARS) models^1^ included: Simple linear model with two predictors; both lin-log models (predictors log transformed) or log-lin models (dependent variable log transformed, or both (power function); models where the two predictor variables are combined (CV models); and model with interaction-term models (INT model); and finally, we included INT models excluding sampling as a separate predictor variable (noSE).

Model ID	Model	Model type
AFTrilm1	*f*(*SR*) = *β*_*0*_ +*β*_*1*_*A + β*_*2*_*SE*	simple linear no-INT
AFTrilm2	log *f*(*SR*) = *β*_*0*_ *+ β*_*1*_log*A* + *β*_*2*_log*SE*	Log-log no-INT
AFTrilm3	*f*(*SR*) = *β*_*0*_ + *β*_*1*_log*A* + *β*_*2*_*SE*	lin-log no-INT
AFTrilm4	log *f*(*SR*) = *β*_*0*_ *+ β*_*1*_log*A* + *β*_*2*_*SE*	log-lin no-INT
AFTrilm5	*f*(*SR*) = *β*_*0*_*+ β*_*1*_log*A* + *β2SE*	lin-log no-INT
AFTrilm6	log *f*(*SR)* = *β*_*0*_ *+ β*_*1*_*A* + *β*_*2*_log*SE*	log-lin no-INT
AFTrilm7	*f*(*SR*) = *β*_*0*_ + *β*_*1*_*A + β*_*2*_log*SE*	lin-log no-INT
AFTrilm8	log *f(SR) = β*_*0*_ *+ β*_*3*_(log*A*)(log*SE*)	power CV model
AFTrilm9	*f(SR) = β*_*0*_ *+ β*_*3*_(log*A*)(log*SE*)	lin-log CV model
AFTrilm10	log *f(SR) = β*_*0*_ *+ β*_*3*_(*A*)(log*SE*)	log-lin CV model
AFTrilm11	*f(SR) = β*_*0*_ *+ β*_*3*_(*A*)(log*SE*)	lin-log CV model
AFTrilm12	log *f(SR) = β*_*0*_ *+ β*_*3*_(log*A*)(*SE*)	log-lin CV model
AFTrilm13	*f(SR) = β*_*0*_ *+ β*_*3*_(log*A*)(*SE*)	lin-log CV model
AFTrilm14	*f(SR) = β*_*0*_ *+ β*_*3*_(*A*)(*SE*)	CV model
AFTrilm15	log *f*(*SR*) = *β*_*0*_ *+ β*_*1*_log*A + β*_*2*_log*SE + β*_*3*_(*logA*)(*logSE*)	power INT
AFTrilm16	*f*(*SR*) = *β*_*0*_ *+ β*_*1*_log*A* + *β*_*2*_log*SE* + *β*_*3*_(*logA*)(*logSE*)	lin-log INT
AFTrilm17	log *f*(*SR*) = *β*_*0*_ *+ β*_*1*_log*A + β*_*2*_*SE + β*_*3*_(*logA*)(*SE*)	log-lin INT
AFTrilm18	*f*(*SR*) = *β*_*0*_ *+ β*_*1*_log*A + β*_*2*_*SE* + *β*_*3*(_*logA*)(*SE*)	lin-log INT
AFTrilm19	log *f*(*SR*) = *β*_*0*_ *+ β*_*1*_*A* + *β*_*2*_log*SE* + *β*_*3*_(*A*)(*logSE*)	log-lin INT
AFTrilm20	*f*(*SR*) = *β*_*0*_ *+ β*_*1*_*A + β*_*2*_log*SE* + *β*_*3*_(*A*)(*logSE*)	lin-log INT
AFTrilm21	*f*(*SR*) = *β*_*0*_ *+ β*_*1*_*A + β*_*2*_*SE* + *β*_*3*_(*A*)(*SE*)	INT
AFTrilm22	log *f*(*SR*) = *β*_*0*_ *+ β*_*1*_log*A* + *β*_*3*_(*logA*)(*logSE*)	power INT noSE
AFTrilm23	*f*(*SR*) = *β*_*0*_ *+ β*_*1*_log*A* + *β*_*3*_(*logA*)(*logSE*)	lin-log INT noSE
AFTrilm24	log *f*(*SR*) = *β*_*0*_ *+ β*_*1*_log*A* + *β*_*3*_(*logA*)(*SE*)	log-lin INT noSE
AFTrilm25	*f*(*SR*) = *β*_*0*_ *+ β*_*1*_log*A* + *β*_*3*_(*logA*)(*SE*)	lin-log INT noSE
AFTrilm26	log *f*(*SR*) = *β*_*0*_ *+ β*_*1*_*A + β*_*3*_(*A*)(*logSE*)	log-lin INT noSE
AFTrilm27	*f*(*SR*) = *β*_*0*_ *+ β*_*1*_*A + β*_*3*_(*A*)(*logSE*)	lin-log INT noSE
AFTrilm28	*f*(*SR*) = *β*_*0*_ *+ β*_*1*_*A + β*_*3*_(*A*)(*SE*)	INT noSE

^1^Abbreviations used for species richness (SR), area of the forest remnants (A), and sampling effort (SE).

**Table 3:** List of generalized linear models used for comparison^1^.

Model ID	Model	Model type
AFGAM1	log *f*(*SR*) = *y*_*i* =_ *f*_*1*_ + *f*_*2*_(log*A*) + *f*_*3*_(log*SE*)	log-log GAM
AFGAM2	*f*(*SR*) = *y*_*i* =_ *f*_*1*_ + *f*_*2*_(log*A*) + *f*_*3*_(log*SE*)	semi-log GAM
AFGAM3	log *f*(*SR*) = *y*_*i* =_ *f*_*1*_ + *f*_*2*_(log*A*) + *f*_*3*_(*SE*)	log-log GAM
AFGAM4	*f*(*SR*) = *y*_*i* =_ *f*_*1*_ + *f*_*2*_(log*A*) + *f*_*3*_(*SE*)	semi-log GAM
AFGAM5	log *f*(*SR*) = *y*_*i* =_ *f*_*1*_ + *f*_*2*_(*A*) + *f*_*3*_(log*SE*)	log-log GAM
AFGAM6	*f*(*SR*) = *y*_*i* =_ *f*_*1*_ + *f*_*2*_(*A*) + *f*_*3*_(log*SE*)	semi-log GAM
dAFGAM7	*f*(*SR*) = *y*_*i* =_ *f*_*1*_ + *f*_*2*_(*A*) + *f*_*3*_(*SE*)	GAM

^1^Abbreviations used for species richness (SR), area of the forest remnants (A), and sampling effort (SE).

### Case study dataset: Non-volant small mammals

Non-volant small mammals are good models for questions in landscape ecology, particularly forest fragmentation questions [69], because non-volant small mammals have small home ranges, short lifespans, short gestation periods, high diversity, and limited dispersal abilities compared to larger or volant vertebrates; and they are an important prey base for predators, consumers of invertebrates and vegetation, and consumers and dispersers of seeds and fungi [70].

We used data for non-volant small mammal species from 68 Atlantic Forest remnants from 20 published studies [59,70] conducted throughout the Atlantic Forest in Brazil and Paraguay from 1987 to 2013 to assess the relationships between species richness, sampling effort (i.e. trapnights), and forest remnant area (Fig 1A). We used only sites that had complete data sets for these three variables per forest remnant for the construction of the models. Sampling effort between studies varied from 168 to 31,960 trapnights per remnant. Compiling a matrix of all species found at each site, we then eliminated all large rodents and marsupials (> 1.5 kg) because they are more likely to be captured in Tomahawks (large cage traps), based on personal experience and the average sizes of those animals. Inclusion of large rodents and marsupials highly skewed species richness between studies that did and studies that did not use the large traps; hence, we used only non-volant mammals < 1.5 kg.

In addition to the published studies noted above, we also included data from a sampling expedition by the authors from 2013 from 6 forest remnants from Tapytá Reserve, Caazapá Department, in eastern Paraguay (S1 Table). Sampling effort followed de la Sancha [70] and consisted of Sherman live traps, snap traps, and pitfall traps with drift fences. The overall sampling effort consisted of eight nights, using 15 trap stations with two Sherman and two snap traps per station on four lines per grid (1,920 trapnights), and 7 buckets per pitfall line (56 trapnights), totaling 1,976 trapnights per forest remnant. The data collected in this 2013 study were approved by the Institutional Animal Care and Use Committee (IACUC) at Rhodes College.

Comparative analyses of SARs based on endemic species versus SARs based on generalist species have found estimated species richness patterns to be statistically different, and species curve patterns based on endemic or generalist species to be different in shape [41,49,71]. Furthermore, endemic or specialist species are more prone to local extirpation as a consequence of habitat fragmentation, and therefore amalgamating all species in an assemblage may mask species loss [41]. Instead of running EARs, which are primarily based on power functions, we ran our models with different subsets of the original dataset of species, based on the species’ sensitivity to deforestation. Specialist and generalist species tend to respond differently to habitat changes as many habitat types provide resources used by generalists, therefore loss of one habitat type is not as detrimental to their populations as it may be for species that rely on one specific habitat type. Therefore, we used multiple types of species groups to evaluate potential differences in species richness responses to changes in habitat area. Overall, we analyzed models for the entire assemblage of non-volant mammals < 0.5 kg (which included introduced species), as well as for two additional datasets that were subsets of the entire non-volant mammal assemblage: 1) the native species forest assemblage and 2) the forest-specialist (endemic equivalents) assemblage. The native species forest assemblage consisted of only forest species, with all grassland (e.g., *Calomys tener*) and introduced (e.g., *Rattus rattus*) species eliminated from the dataset. For the forest-specialist assemblage, we took the native species forest assemblage dataset and we eliminated all forest species that have been documented in other non-forest habitat types or agrosystems [72–74], thus leaving only forest specialists. We assumed that forest-specialist species, like endemics, are more sensitive to continued fragmentation and warrant a unique assemblage because it can be inferred that these species will be the most negatively affected by deforestation and potentially go locally extinct. The purpose of the multiple assemblage analyses was to compare the response differences among the entire, forest, and forest-specialist assemblages.

### Traditional species-area functions

We implemented 8 traditional SAR models (Table 1), using data of non-volant small mammals in the Atlantic Forest. There are more than 27 traditional SAR models; however, we restricted our comparison to only 8 functions. SAR models often are represented by a steep increase in species richness as area increases, but then species richness typically reaches an asymptote. Although SAR models can be linearized using log-transformations for visualization and statistical analyses, a true linear relationship has not been shown to be representative for traditional SAR models (see [32,35,75,76]). In our analyses, we included and compared 8 major nonlinear SAR models (power, exponential, negative exponential, monod (convex models), rational, logistic, Lomolino, and cumulative Weibull (sigmoidal models)) functions (Table 1) for the three different datasets of the entire extent of the Atlantic Forest from the 20 studies outlined above. The power and exponential functions lack an asymptote, and the rest of the functions show asymptotes [25]. For the traditional SAR models, regression validations were considered for homoscedasticity using a Pearson’s correlation of the residual magnitude and areas or fitted values. Models that showed significant homoscedasticity, α ≤ 0.05, were considered not valid [25]. We completed all of these analyses using the mmSAR R package [25].

### Sampling effort and species-area relationships (SESARS) models

Several of the SAR models that have been proposed have included using an additional variable for the traditional species-area models [35]. We were interested in various approaches that included linear and non-linear models with two predictor variables. It is clear that larger areas house more species, and increased sampling tends to result in higher species richness. Thus, our first objective was to identify models where both of these predictive variables resulted in significant contributions to the overall model.

We tested 28 linear multivariate models that predict species based on additive and multiplicative relationships of area and sampling with variations of log transformations per variable including power models, combined (CV models), interaction-term models (INT model) [35], with power and semi-log variations (Table 2). We tested 7 non-linear multivariate generalized additive models (GAMs) that predict species richness based the relationship between area and sampling. These models smooth out the relationship between these variables (Table 3). Additive models tend to implement smoothing functions with capture nonlinear relationships between variables [77]. Smoothness controlling estimation was conducted using maximum likelihood (ML); we did not use restricted maximum likelihood (REML), as it does not permit model comparisons [78], see S1 File for details. While there is considerable turnover in species along the Atlantic Forest latitudinal gradient, there does not appear to be geographic structure in functional diversity along this gradient [59, 79], All multivariate analyses were run in R using the packages lme4, MASS, mgcv, mmSAR, and AICcmodavg [23,80,81].

While some authors have argued that comparison between sites requires equal sampling (e.g., equal trapnights, [82]) because it may be otherwise difficult to distinguish between the influence of sampling and the influence of area (or other variables); others have suggested that there should be proportional or nested sampling in accordance to increases in area [18,58]. This is important to disentangle the difference between species-area relationship versus species-sampling relationships, two relationships that are related but not the same (see [18]). However, there is still no consensus about what approach is best or most appropriate for comparative purposes [18]. Our approach is unique and valuable because it permits us to apply both approaches when using the predictive models, given that we were working with a multivariate regression plane.

We considered a model to be robust based on two criteria. First, we tested our models to null models. Doing so ensured that the combination of variables performed better than by a random model, given that it usually helps to validate models. Second, we selected only models where all predictors significantly contributed to the species richness. Those SESARS models that had either 1) both predictive variables as significant or 2) the combination of area and sampling as significant, were compared with the eight major families of traditional species-area models.

Any one dataset can have multiple models that provide valid inference, assuming that datasets and predictive variables of a model are sound. Akaike Information Criterion (AIC) provides model selection that is objective and omnibus [83, 84], thus AIC metrics for traditional SAR models were generated in mmSAR and AIC metrics for all other models were generated using function ‘AIC’ in R (S1 File). The best model was visualized using ‘ggpredict’. SESARS-predicted species richness, along with standard error values, were calculated using the R function ‘predict’ (Fig 1B).

### Geospatial analysis for area

We used Hansen et al. [68] data (updated for 2014; http://earthenginepartners.appspot.com/science-2013-global-forest/download_v1.2.html) to obtain raster files of forest cover in 2000 and forest loss as of 2014. We created a mosaic of the raster files, and then took the 2000 forest cover data and subtracted the raster files of the deforestation data from 2014 deforestation data to obtain the estimated 2014 forest cover. The 2014 forest data were clipped to fit the extent of the Atlantic Forest, using the map from [85] as a reference. We then extracted only the data from Paraguay. The data were projected to South America Albers Equal Area Conic. We then converted the raster data into a shapefile representing the Atlantic Forest in Paraguay. We calculated the area of each feature (forest remnant) and then extracted forest remnants that were 0.50 ha and larger for use in the analyses. All spatial analyses were conducted using ArcGIS 10.1. These area metrics became our area values to include in our predictive model (Fig 1C).

### Trapping effort estimation

The multivariate models we developed permitted us to include any sampling effort we decided upon as function of our three dimensions. We could have used the same sampling effort for all remnants, for example, or we could have included sampling effort that was “proportional” to area. Making proportional estimations of sampling to implement in a predictive model is complicated. The approach we opted for was to calculate an appropriate sampling metric that had meaning based on our original empirical data. We estimated sampling effort using the linear relationship between area and sampling of the original empirical data, via a log-log regression. This provided an unbiased estimate of sampling, and it was proportional to that used along the entire Atlantic Forest by other researchers (S1 Table). This allowed us to estimate an adequate sampling effort for each of the forest remnants of eastern Paraguay. These values of area and sampling were then implemented in the best-fit multivariate model to predict species richness for all of eastern Paraguay (Fig 1D).

### Species estimates in eastern Paraguay

Finally, we included the area of the individual forest remnants of eastern Paraguay (Fig 1C) and the estimated corresponding proportional trapping effort (Fig 1D) in the best-fit species predictive model (Fig 1E). Predicted species richness for each assemblage model was compared and significance was tested via permutation tests. The permutation began with a comparison of observed mean difference between pairwise comparisons between assemblages. For each pairwise comparison a null distribution of mean differences was developed by changing the species richness per site via permutation for 10,000 replications. P-values were then estimated as the number of observations equal to or more extreme than the original observed mean differences. This permitted us to test that there were significant differences between assemblages based on functionality. Code for running the permutation test was developed by us and run on R. Estimated species richness from the best-fit model was then spatially modeled for all remnants in eastern Paraguay that were 0.50 ha and larger (Fig 1F). We did so for all three assemblages: entire assemblage, native species forest assemblage, and forest-specialist assemblage.

## Results

### Best-fit model comparisons for the Atlantic Forest

We identified all of the models where all of their included parameters included were significantly contributing to the SESAR (entire assemblage: S2 Table; native species forest assemblage: S3 Table; and forest specialist assemblage: S4 Table). For the entire small mammal assemblage, we identified 11 combined or interaction-term SESAR models where all the parameters included, demonstrated significant contributions to the SESAR (S2 Table); and 9 combined or interaction-term SESAR models the native species forest assemblage, (S3 Table); and two SESARS models for the forest-specialist assemblage (S4 Table). None of the generalized additive models (GAMs) showed significant contribution by both area and sampling (S5–S7 Tables) for any of the assemblages. Sampling effort into consideration improved our models, compared to the traditional species-area models (Tables 4 and 5). All best-fit models were robust as these outperformed null models and all predictors significantly contributed to species richness (S5 and S6 Tables). The power-law INT models that excluded sampling as an independent variable were the most robust for the entire assemblage (Trilim22 *P* < 0.0001, F-value = 21.36_*2*,*64*_, Adj. R^2^ = 0.38 [log *f(SR)* = *β*_0_ + *β*_1_log*A* + *β*_3_(log*A*)(log*SE*)], Table 4) and native species forest assemblage (Trilim22_For, *P* < 0.0001, F-value = 13.71_*2*,*64*_, Adj. R^2^ = 0.28 [log *f(SR)* = *β*_0_ + *β*_1_logA + *β*_3_(logA)(logSE)], Table 5). Meanwhile, for the forest-specialist species, the logistic species-area function was the best-fit; however, the power, expo and ratio traditional species-area functions were just as valid (Table 6). The logistic model indicated that there was no correlation between the residual magnitude and areas (Pearson's r = 0.138, and *P* = 0.27) which indicatives a valid model (valid models should be nonsignificant for this analysis). Other parameters of the logistic species-area model included c = 4.99, z = 0.00008, f = -0.081. However, the power, exponential, and rational models were just as likely to be valid with ΔAIC less than 2 (Table 6); and these models did not exhibit correlations between variables (Pearson's r = 0.14, and *P* = 0.27; r = 0.14, and p = 0.28; r = 0.15, and *P* = 0.23). Other parameters were as follows: power, c = 1.953 and z = 0.068; exponential c = 1.87 and z = 0.192; and rational c = 2.300, z = 0.0004, and f = 0.00008.

**Table 4:** Best-fit sampling effort and species-area relationships models identified for the entire assemblage of non-volant small mammals in the Atlantic Forest after comparison of 18 linear, 6 generalized linear model models, and 8 traditional species-area models. Tables 2–5 outline the models.

Entire Models	AIC	Δi AIC*	Log L	wi
TriLm22	88.3	0.00	1.00000	0.96416
TriLm17	95.1	6.86	0.03237	0.03121
TriLm10	99.7	11.43	0.00330	0.00318
TriLm24	101.4	13.12	0.00141	0.00136
TriLm8	108.0	19.73	0.00005	0.00005
TriLm26	109.2	20.89	0.00003	0.00003
TriLm9	111.3	23.02	0.00001	0.00001
Null	118.5	30.25	0.00000	0.00000
Logist	187.8	99.55	0.00000	0.00000
Ratio	188.6	100.31	0.00000	0.00000
Power	190.6	102.31	0.00000	0.00000
Expo	191.5	103.22	0.00000	0.00000
Weibull	192.6	104.31	0.00000	0.00000
Lomolino	192.6	104.34	0.00000	0.00000
NegExpo	197.6	109.39	0.00000	0.00000
Monod	260.7	172.47	0.00000	0.00000
TriLm23	349.7	261.46	0.00000	0.00000
TriLm21	360.2	271.94	0.00000	0.00000
TriLm13	362.7	274.43	0.00000	0.00000
TriLm25	364.6	276.35	0.00000	0.00000
TriLm11	368.9	280.60	0.00000	0.00000
TriLm12	376.6	288.37	0.00000	0.00000
TriLm14	377.1	288.86	0.00000	0.00000

*Based on criteria *sensu* Burnham and Anderson [83], **Δ**_***i***_ AIC values < 2 are indicative of substantial evidence for model validity, **Δ**_***i***_ values of 3 to 7 offer less support, and **Δ**_***i***_ values > 10 indicate very unlikely evidence for those models.

**Table 5:** Best-fit sampling effort and species-area relationships models identified for the native species forest assemblage of non-volant small mammals in the Atlantic Forest after comparison of 28 linear, 7 generalized linear model models, and 8 traditional species-area models. Tables 2 and 4 outline the models.

Forest Models	AIC	Δi AIC*	Log L	wi
TriLmFor22	101.3	0.00	1.00000	0.88814
TriLmFor17	105.7	4.37	0.11230	0.09974
TriLmFor10	111.0	9.67	0.00794	0.00705
TriLmFor24	113.0	11.67	0.00293	0.00260
TriLmFor8	113.3	11.98	0.00250	0.00222
TriLmFor9	118.1	16.74	0.00023	0.00021
Null	121.2	19.89	0.00005	0.00004
Logist	179.6	78.30	0.00000	0.00000
Power	180.5	79.16	0.00000	0.00000
Ratio	180.6	79.23	0.00000	0.00000
Expo	181.1	79.71	0.00000	0.00000
Weibull	182.5	81.16	0.00000	0.00000
NegExpo	186.2	84.87	0.00000	0.00000
Monod	252.0	150.66	0.00000	0.00000
TriLmFor23	351.6	250.27	0.00000	0.00000
TriLmFor18	359.0	257.67	0.00000	0.00000
TriLmFor21	359.6	258.28	0.00000	0.00000
TriLmFor11	362.2	260.86	0.00000	0.00000
TriLmFor13	363.2	261.87	0.00000	0.00000
TriLmFor12	370.6	269.27	0.00000	0.00000
TriLmFor14	372.3	270.98	0.00000	0.00000

*Based on criteria *sensu* Burnham and Anderson [83], **Δ**_***i***_ AIC values < 2 are indicative of substantial evidence for model validity, **Δ**_***i***_ values of 3 to 7 offer less support, and **Δ**_***i***_ values > 10 indicate very unlikely evidence for those models.

**Table 6:** Best-fit sampling effort and species-area relationships models identified for the forest-specialist assemblage of non-volant small mammals in the Atlantic Forest after comparison of 21 linear, 7 generalized linear model models, and 8 traditional species-area models. Tables 2 and 4 outline the models.

Endemic Models	AIC	Δi AIC*	Log L	wi
Logist	123.6	0.00	1.00000	0.27093
Power	123.8	0.16	0.92537	0.25071
Expo	124.1	0.47	0.79242	0.21469
Ratio	125.2	1.53	0.46423	0.12577
Weibull	125.8	2.16	0.34042	0.09223
NegExpo	127.2	3.56	0.16854	0.04566
TriLmEnd8	171.7	48.01	0.00000	0.00000
TriLmEnd22	172.8	49.17	0.00000	0.00000
Null	174.1	50.44	0.00000	0.00000
TriLmEnd26	176.3	52.68	0.00000	0.00000
Monod	176.8	53.15	0.00000	0.00000
TriLmEnd11	311.4	187.72	0.00000	0.00000

*Based on criteria *sensu* Burnham and Anderson [83], **Δ**_***i***_ AIC values < 2 are indicative of substantial evidence for model validity, **Δ**_***i***_ values of 3 to 7 offer less support, and **Δ**_***i***_ values > 10 indicate very unlikely evidence for those models.

### Species richness in the Atlantic Forest remnants of Paraguay

**Fig 2:**
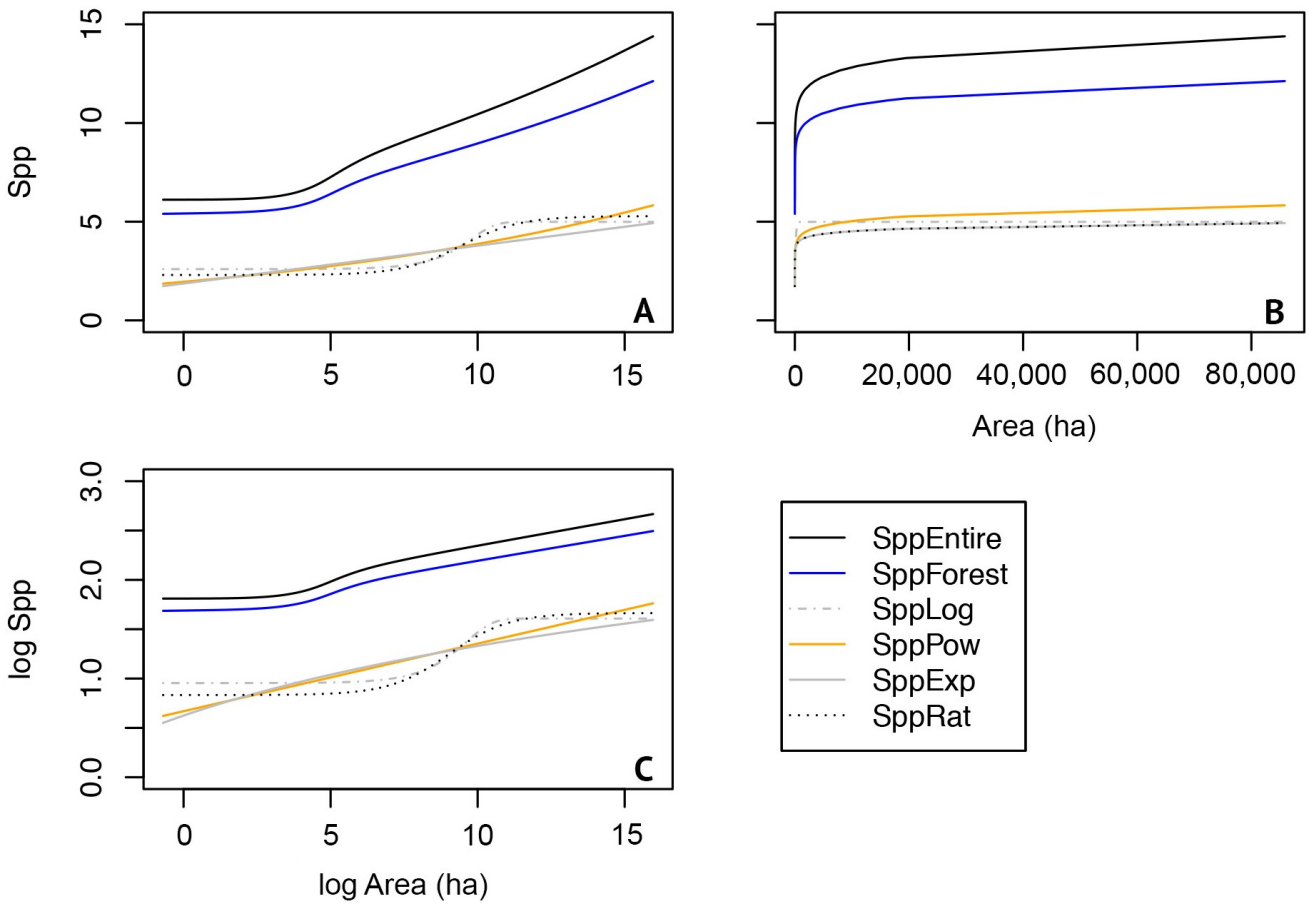
Based on Akaike Information Criterion (AIC), models with values lower than Δi AIC of 2 were just as likely to be valid (see Tables 4–6). Plots represent the best-fit models for the entire assemblage of small mammals (SppEntire), the native species forest (SppForest), and four species-area models for forest specialists (logistic: SppLog, power: SppPow, exponential: SppExp, and ratio: SppRat). The plots show A) log-area and predicted species relationships; B) area and predicted species richness relationships, which show that most species accumulations were reached at relatively small forest areas; and C) the log area and log species relationships that are valuable for comparison of patterns of species accumulations. This suggests that while the largest forest remants have the highest species richness, small- and medium-sized remnants are valuable for conservation efforts from the perspective of small mammals.

Assemblage specific models varied in the predicted species richness based on their sensitivity to deforestation. Pair-wise permutation tests comparing predicted species richness between the entire small mammal assemblage, native species forest assemblage, and forest-specialist assemblage for the Atlantic Forest of Paraguay were highly significant (*P* < 0.0001; Fig 2). SESARS models for the entire and native species forest assemblages showed parallel patterns and, as expected, the entire assemblage showed consistently higher species richness throughout the region. All models clearly showed sigmoidal relationships even when log-transforming area and species (Fig 2). For Atlantic Forest remnants in Paraguay that were 0.50 ha and greater, species richness estimates varied from 6 to 12 species for the entire assemblage, 5 to 10 species for the native species forest assemblage, and 2 to 5 species for the forest-specialist assemblage (including all the likely models: logistic, power, exponential, and rational). Species richness and area plots of the raw data showed that most of the species accumulations appeared at relatively small areas (Fig 2C) and with different hypothetical sampling efforts for the entire assemblage and native species forest assemblage, and most species accumulation was reached for forest specialists when forest area was considerably larger.

**Fig 3:**
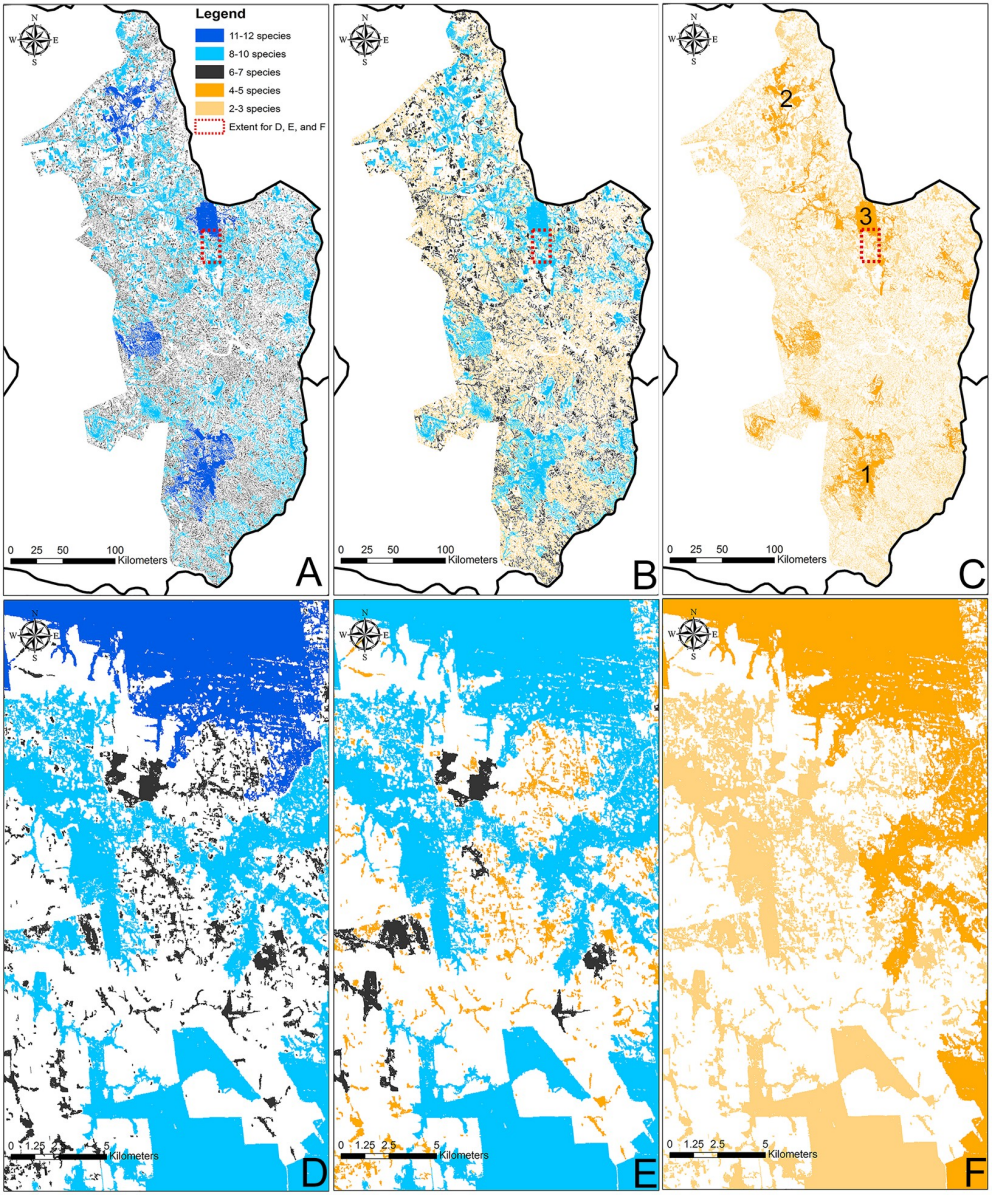
The Atlantic Forest in Paraguay primarily consists of forest remnants that are 50 ha and smaller. Maps identify species richness remnants with high species richness for non-volant small mammals based on (A) predictive SESARS for the entire non-volant, small mammal assemblage; B) SESARS for the native species forest assemblage; and C) ratio species-area model for the forest-specialist assemblage, with the three largest remnants noted in order of size (1–3). Species richness among the three assemblages varied from (D) 6–12 species for the entire assemblage, to (E) 5–10 species for the native species forest assemblage, to (F) 2–5 species for the forest-specialist assemblage.

Our geospatial analysis recovered 140,913 Atlantic Forest remnants that were 0.50 ha and larger in Paraguay. Visualization of species richness in the Paraguayan Atlantic Forest landscape produced very similar patterns, regardless of species groups used, predictions recovered the same forests remnants as most species rich (Fig 3A–3C). The number of predicted species were significantly different between assemblages (Fig 3D–3F) and supported with the spatial models. As expected, the estimated species richness varied more for the entire assemblage and the least for the forest-specialist assemblage. The 15 remnants in Paraguay that were larger than 15,844 ha were predicted to have a maximum of 5 forest-specialist species each. However, the same forest remnants had much greater predicted species richness when the entire and native species forest assemblages were modeled (Fig 2; [10]). Of the 140,913 Atlantic Forest remnants that were 0.50 ha and larger in Paraguay, 140,898 remnants (99.99%) were predicted to have 2–3 species for the forest-specialist assemblage, and species richness for the entire assemblage and the native species forest assemblage was predicted to be 5 or greater for 100% of the forest remnants (Fig 3A and 3B). Even so, species richness for the entire assemblage and the native species forest assemblage was ≤ 6 for 99.52% and 98.51% of the forest remnants, respectively (S8 Table).

## Discussion

### Multivariable models

As the impacts of increasing human populations and activity accelerate and increase in both scope and intensity, many regions of the world might not have had the luxury of time and resources to amass large datasets to model the impacts of habitat loss and fragmentation on biodiversity and conservation. While perhaps over-simplistic, we can pool smaller studies (many of which might have variable sampling efforts as they come from different authors) to address the impact of human land cover changes. The SESARS approach allows us to incorporate both the sampling efforts and area with flexibility and robustness, and this approach is particularly valuable for predictive modeling. Many variables other than area can affect species richness estimates in forest remnants [13, 14, 35, 73, 74], and one of the most obvious and routinely overlooked is sampling effort [58]. Our analysis corroborates that different assemblages do not have the same responses to habitat fragmentation, i.e. the entire assemblage versus forest-specialist species. Therefore, use of SARs for modeling should consider various sampling efforts, and multiple assemblages, when used for conservation of management efforts.

### SESARS models and spatial analyses

Coupled with geospatial analysis, SESARS models are valuable for conservation and management alike. First, although our assemblages varied in predicted species richness, once they were spatially modeled there were clear spatial patterns that highlighted species-rich regions or remnants that are of potential conservation value. Second, our framework can be expanded to species assemblages or guilds that are more appropriate to the question at hand and can also be used at any spatial scale from local to regional to global, as well as to any land cover type. Third, we were able to identify medium-size forest remnants (e.g., in the northernmost portion of the Atlantic Forest of Paraguay) that have potential conservation value based on the species richness of all three different assemblages (Fig 2). The scanning of maps alone tends to bias observers to identify large, strangely shaped, or very isolated remnants. Our analyses allowed us to highlight seemingly unimportant remnants that were in fact potential conservation priority areas, based on the estimated richness visualized by our results. Had we used maps without richness visualizations, these potentially important forest remnants may have been overlooked. Our findings suggest small remnants are valuable and help to house some species and mitigate local extinctions. Although, it is worth noting that our that dataset might actually reflect patterns of populations yet to experience extinction debt [86]. Or it may be the case that the area that would reflect local extinction tends not be sampled and not included in our model (i.e. less than 0.50 ha). In addition, our model only applied to small mammals, and larger-bodied fauna may be more sensitive to extinction [87]. Although, empirical studies have found negative, positive, and no relationships for area, body size, and extinction [88]. Fourth, this model has great potential in forecasts. Our SESAR approach is robust in predicting species richness for areas based on future forest loss. Lastly, perhaps the most valuable aspect of our approach is the ability to integrate studies of varying sampling effort. Currently, there is still no consensus about the best or most appropriate scheme for sampling as a function of area [18]. Our SESAR approach allows users to explore either or both of these sampling schemes as needed for their available datasets. Given the logistical complexities of sampling in remote regions and the cost of long-term expeditions, even small campaigns with modest sampling efforts can add value to the modeling of broad scale patterns using our SESAR approach. Among the benefits of having different sampling efforts is that one can anticipate field work effort for sampling different sized remnants and thus logistical resources (e.g. funding, field work hours needed) can be prioritized as needed. Furthermore, as newer and additional data are collected, the model can be improved even if the data originate from different sources with varying sampling efforts.

### Paraguay’s Atlantic Forest

The Atlantic Forest in South America is a biodiversity hotspot [89], but much of it has been deforested, including in Paraguay in recent years [17,90]. For each of the three assemblages of nonvolant small mammals (entire, native species forest, and forest-specialist), the largest forest remnants were predicted to have the greatest species richness, as expected. Although the forest-specialist assemblage had a maximum species richness of 5 for the Paraguayan forest remnants, and only 7 forest remnants had this maximum number of 5 species, the forested area that comprised these 7 remnants totaled 32.71% of the entire Atlantic Forest in Paraguay. While the largest forest remnants had the greatest species richness regardless of assemblage, small- (< 125 ha) and medium-sized (~15,000 ha) remnants still maintained 5–10 species when examining the entire and native species forest assemblages. These findings highlight the importance of small and medium remnants for small mammal conservation. In Paraguay, it is difficult to make the case that there are endemic Atlantic Forest species per se, which is why we used the term forest specialist. That said, it is important to note that at least 30 new species records have been documented for Paraguay since 2002, and the taxonomy for mammals is still very unclear, even for megafauna [91]. Furthermore, new species records are validating our models, for example, *Juliomys pictipes*, a particularly rare Atlantic Forest species, was first documented in Paraguay 2009 [92] and more records are being added [70]. More recently Atlantic Forest endemics *Delomys dorsalis* and *Abrawayaomys ruchii* have been recently discovered in the country [93, 94, 95]. It is very likely that new species will be found in these larger forest remnants with continued field expeditions [6] and improved taxonomic and collections studies [95].

The two largest forest “remnants” in Paraguay were expanses of patchwork forest surrounded by a non-forest matrix, but in reality, these larger forest remnants likely consist of multiple remnants that are separated by short (< 50 m) distances. As a result, 30-m resolution satellite imagery, which is the basis of the forest cover data from [68] and the basis for many studies of deforestation [96], may overestimate connectivity in the landscape. Although 30-m resolution satellite imagery is common for analyzing larger areas, smaller-resolution imagery can often detect patch size, shape, and connectivity better [97]; however, such imagery comes at a financial cost, a time cost to analyze the data, and limitations for processing such large quantities of data [98]. Furthermore, given that anthropogenic disturbances in a forest can also contribute greatly to biodiversity loss [99], the linear, sinewy forest remnants with high edge-to-area ratios may have lower species richness than forest remnants that are of the same size but more intact. We believe that this framework can be easily replicated for any fragmented landscape, archipelago, or sky islands system, where datasets are limited and where empirical data from many authors with different sampling effort; and may provide more informative predicted species models. With our models, we are able to find the regions with the highest richness, but we can potentially also identify the areas more susceptible to fauna loss and subsequently focus efforts on the conservation of these sites. Furthermore, this approach can be applied immediately, which is important given the logistical difficulties of sampling at multiple biogeographical scales, the limitations of sampling in inaccessible and remote locations, and the current and intensifying rates of global deforestation. This approach also permits null models that help to prioritize regions to be sampled and regions which may be important richness hotspots. This is valuable where resources are limited for extensive field data collection and where the rates of deforestation are very high and immediate action is important.

### Concluding remarks

Lastly, our initial analysis showed that the power function may not always be best approach to understanding SARs and we should expand our horizons to include additional SAR models as needed. While we did not find a universal function, we did find that incorporating sampling into SAR models can be valuable. Furthermore, there may be additional variable transformations which may optimize these relationships. Thus, this is just the beginning of the possibilities to these models. While SARs may overestimate extinction loss, overestimation is a preferable error over underestimation, and we think that SARs are still valuable for modeling responses of species richness to deforestation and habitat loss and will remain for the foreseeable future. Furthermore, it would be interesting to implement these models when studying true islands and sky islands and implement these models when using other species-area datasets with varying sampling efforts, so comparisons can be made to traditional predictions.

## Supporting information

S1 File Supplementary information for this article contained in this file includes additional statistical methodology details. (DOCX)

S1 Table List of sites in Brazil (BR) and Paraguay (PY) used to develop the predictive species-area relationship (SAR) models with corresponding data, including latitude and longitude of remnant (when available) or location (as documented in study); citations of study; trapnights per remnant as specified by study; area of individual remnant, and corresponding species richness (SR) of all small mammals captured (entire assemblage), forest only species (native species forest assemblage), and forest specialists (forest-specialist assemblage). (DOCX)

S2 Table Results of multiple regression analyses for predicting species richness *f*(*SR*) for the entire non-volant small mammal assemblage in Atlantic Forest remnants using 18 models that included both area of the forest remnants (*A*) and sampling effort (*SE*) of the field studies. (DOCX)

S3 Table Results of multiple regression analyses for predicting species richness *f*(*SR*) for the forest species of non-volant small mammals in Atlantic Forest remnants using 18 models that included both area of the forest remnants (*A*) and sampling effort (*S*) of the field studies. (DOCX)

S4 Table Results of multiple regression analyses for predicting species richness *f*(*SR*) for the endemic/forest specialist species of non-volant small mammals in Atlantic Forest remnants using 18 models that included both area of the forest remnants (*A*) and sampling effort (*S*) of the field studies. (DOCX)

S5 Table Generalized additive model (GAM) results for species richness (*SR*) of the entire assemblage of non-volant small mammals in the Atlantic Forest. The predictors are smoothers for forest remnant area (*A*) and sampling effort (*SE*), with estimated degrees of freedom (e.d.f).
(DOCX)

S6 Table Generalized additive model (GAM) results for species richness (*SR*) of the native species forest assemblage of non-volant small mammal assemblage in the Atlantic Forest. The predictors are smoothers for forest remnant area (*A*) and sampling effort (*S*), with estimated degrees of freedom (e.d.f.).
(DOCX)

S7 Table Generalized additive model (GAM) results for species richness (*SR*) of the forest-specialist assemblage of non-volant small mammals in the Atlantic Forest. The predictors are smoothers for forest remnant area (*A*) and sampling effort (*S*), with estimated degrees of freedom (e.d.f.).
(DOCX)

S8 Table Predicted species richness of non-volant small mammals in Paraguayan Atlantic Forest remnants varies based on size of the forest remnant and the species assemblage (entire, native species forest or forest-specialist). The maximum forest remnant size was 184,553.80 ha.
(DOCX)
