# Supplementary material for: Predictive sampling effort and species-area relationship models for estimating richness in fragmented landscapes
Source: PLoS One. 2019 Dec 31;14(12):e0226529. doi: 10.1371/journal.pone.0226529 (PMC6938349; doi:10.1371/journal.pone.0226529)
Supplement: S1 File — (DOCX) [file pone.0226529.s001.docx]

**Supplementary Information for:** Predictive Sampling Effort and Species-Area Relationship Models for Estimating Richness in Fragmented Landscapes

**Supporting Methods**

**Sampling Effort and Species-Area Relationships (SESARS) models**

**Multivariate Linear Models**

We tested 28 linear multivariate models that predicted species based on additive and multiplicative relationships of area and sampling with variations of log transformations of each variable (S3 Table). Our first approach included a simple linear model with two predictors [35]. Changing the weighting of variables via transformations can change and improve the shape of models, and such transformations are commonly necessary for linear models. Thus, we compared models where one or two of the predictors were log transformed but not the dependent variable (i.e. lin-log models), and models where the dependent variable (species richness) was log transformed (log-lin models). In the case where all variables are log transformed, these take a power function form identified as power. The second approach included models where the two predictor variables are combined (CV models). In this case both predictor and independent variables can be log transformed. The third approach we used included interaction-term models (INT model), which are helpful when one expects the effort of one variable will vary with the second variable; as would be the case if species richness were to increase faster with area as species are sampled over longer periods of time [35]. With this approach, additional interacting terms are included as a separate multiplying parameter [35]. For this we implemented models that were constructed using area and sampling effort plus the multiplier of these two; and models that included area and the multiplier of area*sampling effort only, excluding sampling as an independent predictor from the model. And finally, we compared a power version of the INT model, where the dependent variable was also log transformed [35]. We included various permutations of each of the linear, INT, and power-law INT versions of these models, where we also tested semi-log models (where only one of the predictor variables was log-transformed), in order to test if the fit to the models improved (S3 Table).

**Generalized Additive Models**

We tested 7 non-linear multivariate generalized additive models (GAMs) that predict species richness based the relationship between area and sampling. These models smooth out the relationship between these variables (S4 Table). Species area models are well known to be non-linear [23,32,35]. Additive models tend to implement smoothing functions with capture nonlinear relationships between variables [77]. Therefore, we applied generalized additive model (GAM) approaches with the same predictive variables and transformation variations. We compared these to 6 GAMs with species richness as a function of area plus sampling (S4 Table). We performed the smoothing with a smoothing function ‘s’ in R. This GAM approach only allowed for the addition of predictive models, not the multiplication or division of predictive models. All statistical analyses were evaluated at α = 0.05 for the fitness of the overall model, and we evaluated the significance per coefficient via a T-test. All models were performed based on a Gaussian family distributions and identity link function. For GAM, smoothness controlling estimation was conducted using maximum likelihood (ML); we did not use restricted maximum likelihood (REML), as it does not permit model comparisons [78] .

The Atlantic Forest can be split into at least 4 major metacommunities in the case of small mammals, including a Pernambuco region, a Bahia region, a Southeastern Coast region, and the Interior Atlantic Forest of Paraguay and Argentina [59]. While there is considerable turnover in species along the Atlantic Forest latitudinal gradient, there does not appear to be geographic structure in functional diversity along this gradient [79], suggesting that as species are replaced, they occupy the same niches within the regions of this forest system where they are found. Thus, we assumed that species-area relationships are functionally similar in forest remnants along the entire gradient, independent of centers of endemism or metacommunities. All multivariate analyses were run in R using the packages lme4, MASS, mgcv, mmSAR, and AICcmodavg [23,80,81].

**Akaike Information Criterion (AIC)**

Any one dataset can have multiple models that provide valid inference, assuming that datasets and predictive variables of a model are sound. Akaike Information Criterion (AIC) provides model selection that is objective and omnibus [83, 84], thus AIC metrics for traditional SAR models were generated in mmSAR and AIC metrics for all other models were generated using function ‘AIC’ in R. Comparison to find the best-fit model was selected based on criteria *sensu* Burnham and Anderson [83], where ∆***_i_*** AIC values < 2 were indicative of substantial evidence for model validity, **∆*_i_*** values of 3 to 7 offered less support, and **∆*_i_*** values > 10 indicated very unlikely evidence for those models. The model with the lowest AIC value and ∆*_i_* AIC below 2 was selected as the best fit model was implanted to predict species richness per assemblage. The best model was visualized using ‘ggpredict’. SESARS-predicted species richness, along with standard error values, were calculated using the R function ‘predict’ (Fig 1B).
