# Supplementary material for: Predictive sampling effort and species-area relationship models for estimating richness in fragmented landscapes
Source: PLoS One. 2019 Dec 31;14(12):e0226529. doi: 10.1371/journal.pone.0226529 (PMC6938349; doi:10.1371/journal.pone.0226529)
Supplement: S1 Table — (DOCX) [file pone.0226529.s002.docx]

**S1 Table. List of sites in Brazil (BR) and Paraguay (PY) used to develop the predictive species-area relationship (SAR) models with corresponding data, including latitude and longitude of remnant (when available) or location (as documented in study); citations of study; trapnights per remnant as specified by study; area of individual remnant, and corresponding species richness (SR) of all small mammals captured (entire assemblage), forest only species (native species forest assemblage), and forest specialists (forest-specialist assemblage).**

| **Sites** | **Latitude** | **Longitude** | **Reference** | **Sample Effort** | **Area (Ha)** | **SR Entire** | **SR Forest** | **SR Specialist** |
| --- | --- | --- | --- | --- | --- | --- | --- | --- |
| 1 | -8.25 | -35.084 | Asfora & Mendes Pontes, 2009 | 400 | 6 | 3 | 2 | 2 |
| 2 | -8.25 | -35.084 | Asfora & Mendes Pontes, 2009 | 400 | 50 | 4 | 2 | 1 |
| 3 | -8.25 | -35.084 | Asfora & Mendes Pontes, 2009 | 400 | 38 | 3 | 2 | 1 |
| 4 | -8.25 | -35.084 | Asfora & Mendes Pontes, 2009 | 400 | 118 | 3 | 2 | 1 |
| 5 | -8.71 | -35.834 | Asfora & Mendes Pontes, 2009 | 400 | 50 | 6 | 5 | 3 |
| 6 | -8.71 | -35.834 | Asfora & Mendes Pontes, 2009 | 400 | 50 | 4 | 4 | 3 |
| 7 | -8.71 | -35.834 | Asfora & Mendes Pontes, 2009 | 400 | 300 | 12 | 9 | 6 |
| 8 | -8.71 | -35.834 | Asfora & Mendes Pontes, 2009 | 400 | 500 | 7 | 6 | 5 |
| 9 | -9 | -35.87 | Asfora & Mendes Pontes, 2009 | 400 | 3400 | 4 | 4 | 2 |
| 10 | -9 | -35.87 | Asfora & Mendes Pontes, 2009 | 400 | 50 | 2 | 1 | 1 |
| 11 | -13.525 | -39.035 | Moura, 2003 | 1000 | 3446 | 7 | 7 | 3 |
| 12 | -13.579 | -39.707 | Moura, 2003 | 1000 | 2210 | 7 | 7 | 4 |
| 13 | -13.701 | -39.233 | Moura, 2003 | 1000 | 914 | 8 | 8 | 5 |
| 14 | -13.865 | -39.673 | Moura, 2003 | 1000 | 2025 | 8 | 8 | 3 |
| 15 | -13.953 | -39.451 | Moura, 2003 | 1000 | 1897 | 4 | 4 | 1 |
| 16 | -14.018 | -39.143 | Moura, 2003 | 1000 | 7530 | 2 | 1 | 0 |
| 17 | -14.344 | -39.087 | Moura, 2003 | 1000 | 89 | 3 | 3 | 1 |
| 18 | -14.424 | -39.06 | Moura, 2003 | 1000 | 10224 | 4 | 4 | 2 |
| 19 | -15.155 | -39.527 | Moura, 2003 | 1000 | 2228 | 2 | 2 | 1 |
| 20 | -15.166 | -39.06 | Moura, 2003 | 1000 | 20128 | 5 | 5 | 4 |
| 21 | -15.167 | -39.05 | Pardini, 2004 | 4032 | 7022 | 8 | 8 | 5 |
| 22 | -15.197 | -39.391 | Moura, 2003 | 1000 | 3135 | 4 | 4 | 1 |
| 23 | -15.62 | -39.161 | Moura, 2003 | 1000 | 220 | 3 | 3 | 2 |
| 24 | -15.927 | -39.636 | Moura, 2003 | 1000 | 3215 | 3 | 3 | 2 |
| 25 | -15.967 | -47.95 | Nitikman & Mares, 1987 | 12170 | 4062 | 10 | 9 | 4 |
| 26 | -15.974 | -39.374 | Moura, 2003 | 1000 | 2324 | 2 | 2 | 2 |
| 27 | -16.286 | -39.424 | Moura, 2003 | 1000 | 230 | 3 | 3 | 3 |
| 28 | -16.324 | -39.121 | Moura, 2003 | 1000 | 8090 | 1 | 1 | 0 |
| 29 | -16.512 | -39.304 | Moura, 2003 | 1000 | 17629 | 5 | 5 | 4 |
| 30 | -16.599 | -39.914 | Moura, 2003 | 1000 | 465 | 6 | 6 | 4 |
| 31 | -17.107 | -39.34 | Moura, 2003 | 1000 | 18126 | 2 | 2 | 1 |
| 32 | -17.169 | -39.842 | Moura, 2003 | 1000 | 1450 | 5 | 5 | 3 |
| 33 | -17.292 | -39.673 | Moura, 2003 | 1000 | 574 | 6 | 6 | 3 |
| 34 | -19.397 | -42.301 | Fonseca & Kierulff, 1989 | 9520 | 860 | 10 | 10 | 5 |
| 35 | -19.633 | -42.55 | Fonseca & Kierulff, 1989 | 9520 | 35973 | 14 | 14 | 8 |
| 36 | -19.633 | -42.55 | Stallings, 1989 | 31960 | 35973 | 12 | 12 | 7 |
| 37 | -19.8 | -42.633 | Grelle, 2003 | 5880 | 36000 | 9 | 8 | 6 |
| 38 | -19.805 | -42.642 | Fonseca & Kierulff, 1989 | 9520 | 80 | 8 | 8 | 3 |
| 40 | -20.775 | -40.592 | PaSESARsmani et al., 2004 | 3331 | 390 | 7 | 7 | 5 |
| 41 | -22.5 | -42.25 | Pires et al., 2002 | 3014 | 7 | 9 | 8 | 5 |
| 42 | -22.5 | -42.25 | Pires et al., 2002 | 168 | 1 | 4 | 4 | 3 |
| 43 | -22.5 | -42.25 | Pires et al., 2002 | 977 | 10 | 6 | 6 | 5 |
| 44 | -22.5 | -42.25 | Pires et al., 2002 | 4551 | 9 | 7 | 7 | 6 |
| 45 | -22.5 | -42.25 | Pires et al., 2002 | 1075 | 11 | 4 | 4 | 3 |
| 46 | -22.5 | -42.25 | Pires et al., 2002 | 1220 | 12 | 7 | 7 | 4 |
| 47 | -22.5 | -42.25 | Pires et al., 2002 | 874 | 9 | 7 | 7 | 4 |
| 48 | -22.5 | -42.25 | Pires et al., 2002 | 1618 | 15 | 9 | 8 | 4 |
| 49 | -22.533 | -42.867 | Vieira et al., 2009 | 600 | 10000 | 2 | 2 | 0 |
| 50 | -23.767 | -47.117 | Pardini & Umetzu, 2006 | 8160 | 10700 | 20 | 20 | 13 |
| 51 | -23.767 | -47.117 | Umetsu & Pardini, 2007 | 336 | 9400 | 9 | 9 | 6 |
| 52 | -24.233 | -48.067 | Viveros & Monterio, 2003 | 17361 | 49000 | 20 | 20 | 10 |
| 53 | -24.533 | -47.25 | Bergallo, 1994 | 4704 | 80000 | 6 | 5 | 3 |
| 54 | -26.067 | -48.633 | Quadros et al., 2000 | 1920 | 586 | 12 | 9 | 5 |
| 55 | -24.149 | -55.432 | de la Sancha, 2014 | 31600 | 95758 | 14 | 9 | 3 |
| 56 | -26.459 | -55.758 | de la Sancha, 2014 | 31600 | 77351 | 10 | 6 | 1 |
| 57 | -24.816 | -54.467 | de la Sancha, 2014 | 31600 | 16000 | 15 | 10 | 3 |
| 58 | -24.538 | -55.351 | de la Sancha, 2014 | 25280 | 17507 | 9 | 6 | 2 |
| 59 | -24.713 | -55.438 | de la Sancha, 2014 | 6320 | 1214 | 11 | 8 | 2 |
| 60 | -27.08 | -52.425 | Maestri *et al.* 2014 | 6500 | 300 | 17 | 15 | 6 |
| 61 | -29.1 | -50.05 | Dalmagro & Vieira 2005 | 5178 | 26 | 6 | 5 | 0 |
| 62 | -27.43 | -48.32 | Graipel *et al.* 2006 | 12132 | 1500 | 9 | 8 | 3 |
| 63 | -26.2894 | -55.7507 | Tapyta, Paraguay | 1976 | 1200 | 3 | 3 | 0 |
| 64 | -26.2689 | -55.7889 | Tapyta, Paraguay | 1976 | 633 | 3 | 3 | 0 |
| 65 | -26.2812 | -55.7887 | Tapyta, Paraguay | 1976 | 8 | 5 | 5 | 0 |
| 66 | -26.2689 | -55.7737 | Tapyta, Paraguay | 1976 | 25 | 4 | 4 | 0 |
| 67 | -26.2793 | -55.7761 | Tapyta, Paraguay | 1976 | 2 | 4 | 4 | 0 |
| 68 | -26.2944 | -55.7828 | Tapyta, Paraguay | 1976 | 9 | 5 | 4 | 0 |
