# Supplementary material for: Predictive sampling effort and species-area relationship models for estimating richness in fragmented landscapes
Source: PLoS One. 2019 Dec 31;14(12):e0226529. doi: 10.1371/journal.pone.0226529 (PMC6938349; doi:10.1371/journal.pone.0226529)
Supplement: S2 Table — (DOCX) [file pone.0226529.s003.docx]

**S2 Table. Results of multiple regression analyses for predicting species richness *f*(*SR*) for the entire non-volant small mammal assemblage in Atlantic Forest remnants using 18 models that included both area of the forest remnants (*A*) and sampling effort (*SE*) of the field studies.**

| **Model Name** | **Model** | **Adj R^2^** | **F Stat** | **β_0_ (Intercept)** | **β_1_ (Area)** | **β_2_ (Sampling)** | **β_3_ (Area:Sampling)** |
| --- | --- | --- | --- | --- | --- | --- | --- |
| AFTrilm1 | *f*(*SR*) = *β_0_* + *β_1_A + β_2_SE* | 0.338 | 17.85*_2,64_**** | 5.777*** | -0.000005 | 0.0003*** | - |
| AFTrilm2 | log *f*(*SR*) = *β_0_ + β_1_*log*A* + *β_2_*log*SE* | 0.390 | 22.37*_2,64_**** | -0.29 | -0.0129 | 0.2928*** | - |
| AFTrilm3 | *f*(*SR*) = *β_0_* + *β_1_*log*A* + *β_2_SE* | 0.430 | 26.13*_2,64_**** | -9.02*** | 0.0536 | 2.1221*** | - |
| AFTrilm4 | log *f*(*SR*) = *β_0_ + β_1_*log*A* + *β_2_SE* | 0.290 | 14.48*_2,64_**** | 1.69*** | -0.0096 | 0.00004*** | - |
| AFTrilm5 | *f*(*SR*) = *β_0_+ β_1_*log*A* + *β2SE* | 0.340 | 17.99*_2,64_**** | 5.36*** | 0.0732 | 0.0003*** | - |
| AFTrilm6 | log *f*(*SR)* = *β_0_ + β_1_A* + *β_2_*log*SE* | 0.390 | 22.07*_2,64_**** | -0.32 | -0.0000008 | 0.2864*** | - |
| AFTrilm7 | *f*(*SR*) = *β_0_* + *β_1_A + β_2_*log*SE* | 0.430 | 26.04*_2,64_**** | -8.85** | 0.000004 | 2.14*** | - |
| AFTrilm8 | log *f(SR) =* *β_0_ + β_3_*(log*A*)(log*SE*) | 0.158 | 13.36*_1,65_**** | 1.409*** | - | - | 0.0083*** |
| AFTrilm9 | *f(SR) =* *β_0_ + β_3_*(log*A*)(log*SE*) | 0.115 | 9.70*_1,65_**** | 1.736*** | - | - | 0.000001** |
| AFTrilm10 | log *f(SR) =* *β_0_ + β_3_*(*A*)(log*SE*) | 0.256 | 23.70*_1,65_**** | 1.681*** | - | - | 0.000003*** |
| AFTrilm11 | *f(SR) =* *β_0_ + β_3_*(*A*)(log*SE*) | 0.246 | 22.52*_1,65_**** | 3.482*** | - | - | 0.0754*** |
| AFTrilm12 | log *f(SR) =* *β_0_ + β_3_*(log*A*)(*SE*) | 0.153 | 12.94*_1,65_**** | 6.52*** | - | - | 0.000009*** |
| AFTrilm13 | *f(SR) =* *β_0_ + β_3_*(log*A*)(*SE*) | 0.312 | 30.97*_1,65_**** | 6.096*** | - | - | 0.00003*** |
| AFTrilm14 | *f(SR) =* *β_0_ + β_3_*(*A*)(*SE*) | 0.147 | 12.36*_1,65_**** | 6.772*** | - | - | 0.00000000*** |
| AFTrilm15 | log *f*(*SR*) = *β_0_ + β_1_*log*A + β_2_*log*SE + β_3_*(*logA*)(*logSE*) | 0.390 | 15.32*_3,63_**** | 0.54 | -0.1229 | 0.1788 | 0.0147 |
| AFTrilm16 | *f*(*SR*) = *β_0_ + β_1_*log*A* + *β_2_*log*SE* + *β_3_*(*logA*)(*logSE*) | 0.440 | 18.58_3_*_,63_**** | -0.53 | -1.0730 | 0.9546 | 0.1505 |
| AFTrilm17 | log *f*(*SR*) = *β_0_ + β_1_*log*A* *+ β_2_SE + β_3_*(*logA*)(*SE*) | 0.320 | 11.56*_3,63_**** | 1.49*** | 0.0123 | 0.0001** | -0.00001* |
| AFTrilm18 | *f*(*SR*) = *β_0_ + β_1_*log*A + β_2_SE* + *β_3_*_(_*logA*)(*SE*) | 0.360 | 13.6*_3,63_**** | 4.03** | 0.2147 | 0.0009** | -0.00006 |
| AFTrilm19 | log *f*(*SR*) = *β_0_ + β_1_A* + *β_2_*log*SE* + *β_3_*(*A*)(*logSE*) | 0.390 | 14.84*_3,63_**** | -0.20 | -0.00002 | 0.271*** | 0.000002 |
| AFTrilm20 | *f*(*SR*) = *β_0_ + β_1_A + β_2_*log*SE* + *β_3_*(*A*)(*logSE*) | 0.430 | 17.46*_3,63_**** | -7.98** | -0.0001 | 2.032*** | 0.00002 |
| AFTrilm21 | *f*(*SR*) = *β_0_ + β_1_A + β_2_SE* + *β_3_*(*A*)(*SE*) | 0.356 | 13.16*_3,63_**** | 5.403*** | 0.00005 | 0.0004*** | 0.0000000 |
| AFTrilm22 | log *f*(*SR*) = *β_0_ + β_1_*log*A* + *β_3_*(*logA*)(*logSE*) | 0.380 | 21.36*_2,64_**** | 1.81*** | -0.2627*** | - | 0.0341*** |
| AFTrilm23 | *f*(*SR*) = *β_0_ + β_1_*log*A* + *β_3_*(*logA*)(*logSE*) | 0.440 | 27.07*_2,64_**** | 6.27*** | -1.819*** | - | 0.2540*** |
| AFTrilm24 | log *f*(*SR*) = *β_0_ + β_1_*log*A* + *β_3_*(*logA*)(*SE*) | 0.250 | 11.87*_2,64_**** | 1.75*** | -0.0130 | - | 0.000004*** |
| AFTrilm25 | *f*(*SR*) = *β_0_ + β_1_*log*A* + *β_3_*(*logA*)(*SE*) | 0.300 | 15.3*_2,64_**** | 5.84*** | 0.0451 | - | 0.00003*** |
| AFTrilm26 | log *f*(*SR*) = *β_0_ + β_1_A + β_3_*(*A*)(*logSE*) | 0.160 | 7.063*_2,64_*** | 1.78*** | -0.00006* | - | 0.000007* |
| AFTrilm27 | *f*(*SR*) = *β_0_ + β_1_A + β_3_*(*A*)(*logSE*) | 0.190 | 8.759*_2,64_**** | 6.84*** | -0.00043* | - | 0.00005* |
| AFTrilm28 | *f*(*SR*) = *β_0_ + β_1_A + β_3_*(*A*)(*SE*) | 0.140 | 6.353*_2,64_**** | 6.637*** | 0.00003 | - | 0.000000002 |

P-value significance is identified as follows ‘***’ 0.001 ‘**’ 0.01 ‘*’ 0.05.
