# Supplementary material for: Predictive sampling effort and species-area relationship models for estimating richness in fragmented landscapes
Source: PLoS One. 2019 Dec 31;14(12):e0226529. doi: 10.1371/journal.pone.0226529 (PMC6938349; doi:10.1371/journal.pone.0226529)
Supplement: S3 Table — (DOCX) [file pone.0226529.s004.docx]

**Table S3 Results of multiple regression analyses for predicting species richness *f*(*SR*) for the forest species of non-volant small mammals in Atlantic Forest remnants using 18 models that included both area of the forest remnants (*A*) and sampling effort (*S*) of the field studies.**

| **Model ID** | **Model** | **Adj R^2^** | **F Stat** | **β_0_ (Intercept)** | **β_1_ (Area)** | **β_2_ (Sampling)** | **β_3_ (Area:Sampling)** |
| --- | --- | --- | --- | --- | --- | --- | --- |
| AFTrilm1 | *f*(*SR*) = *β_0_* + *β_1_A + β_2_SE* | 0.192 | 8.839*_2,64_**** | 5.541*** | -0.00001 | 0.0002** | - |
| AFTrilm2 | log *f*(*SR*) = *β_0_ + β_1_*log*A* + *β_2_*log*SE* | 0.330 | 17.41*_2,64_**** | -0.2738 | -0.0114 | 0.2759*** | - |
| AFTrilm3 | *f*(*SR*) = *β_0_* + *β_1_*log*A* + *β_2_SE* | 0.340 | 18.03*_2,64_**** | -6.752** | 0.0462 | 1.7365*** | - |
| AFTrilm4 | log *f*(*SR*) = *β_0_ + β_1_*log*A* + *β_2_SE* | 0.180 | 8.205*_2,64_**** | 1.575*** | -0.0009 | 0.00003*** | - |
| AFTrilm5 | *f*(*SR*) = *β_0_+ β_1_*log*A* + *β2SE* | 0.200 | 9.132*_2,64_**** | 4.87*** | 0.1171 | 0.0002** | - |
| AFTrilm6 | log *f*(*SR)* = *β_0_ + β_1_A* + *β_2_*log*SE* | 0.340 | 17.74*_2,64_**** | -0.4209 | -0.000003 | 0.2893*** | - |
| AFTrilm7 | *f*(*SR*) = *β_0_* + *β_1_A + β_2_*log*SE* | 0.340 | 18.17*_2,64_**** | -7.457** | -0.00001 | 1.885*** | - |
| AFTrilm8 | log *f(SR) =* *β_0_ + β_3_*(log*A*)(log*SE*) | 0.124 | 10.36*_1,65_**** | 1.3441*** | - | - | 0.0076** |
| AFTrilm9 | *f(SR) =* *β_0_ + β_3_*(log*A*)(log*SE*) | 0.060 | 5.191*_1,65_**** | 1.657*** | - | - | 0.026* |
| AFTrilm10 | log *f(SR) =* *β_0_ + β_3_*(*A*)(log*SE*) | 0.154 | 13*_1,65_**** | 1.609*** | - | - | 0.000003*** |
| AFTrilm11 | *f(SR) =* *β_0_ + β_3_*(*A*)(log*SE*) | 0.185 | 15.98*_1,65_**** | 3.5600*** | - | - | 0.0604*** |
| AFTrilm12 | log *f(SR) =* *β_0_ + β_3_*(log*A*)(*SE*) | 0.076 | 6.435*_1,65_**** | 6.089*** | - | - | 0.000006* |
| AFTrilm13 | *f(SR) =* *β_0_ + β_3_*(log*A*)(*SE*) | 0.173 | 14.77*_1,65_**** | 5.782*** | - | - | 0.00002*** |
| AFTrilm14 | *f(SR) =* *β_0_ + β_3_*(*A*)(*SE*) | 0.052 | 4.63*_1,65_**** | 6.301*** | - | - | 0.000000002* |
| AFTrilm15 | log *f*(*SR*) = *β_0_ + β_1_*log*A + β_2_*log*SE + β_3_*(*logA*)(*logSE*) | 0.350 | 11.43*_3,63_**** | -0.3375 | -0.0030 | 0.2846* | -0.0011 |
| AFTrilm16 | *f*(*SR*) = *β_0_ + β_1_*log*A* + *β_2_*log*SE* + *β_3_*(*logA*)(*logSE*) | 0.330 | 11.95*_3,63_**** | -4.1215 | -0.3027 | 1.3749 | 0.0466 |
| AFTrilm17 | log *f*(*SR*) = *β_0_ + β_1_*log*A* *+ β_2_SE + β_3_*(*logA*)(*SE*) | 0.240 | 7.952*_3,63_**** | 1.308*** | 0.0276 | 0.0002** | -0.00001* |
| AFTrilm18 | *f*(*SR*) = *β_0_ + β_1_*log*A + β_2_S* + *β_3_*_(_*logA*)(*SE*) | 0.240 | 8.128*_3,63_**** | 3.276** | 0.2869 | 0.0010** | -0.00008* |
| AFTrilm19 | log *f*(*SR*) = *β_0_ + β_1_A* + *β_2_*log*SE* + *β_3_*(*A*)(*logSE*) | 0.330 | 11.65*_3,63_**** | -0.4389 | 0.00000001 | 0.2915*** | -0.0000003 |
| AFTrilm20 | *f*(*SR*) = *β_0_ + β_1_A + β_2_*log*SE* + *β_3_*(*A*)(*logS*) | 0.330 | 11.96*_3,63_**** | -7.758** | 0.00004 | 1.923*** | -0.00001 |
| AFTrilm21 | *f*(*SR*) = *β_0_ + β_1_A + β_2_SE* + *β_3_*(*A*)(*S*) | 0.238 | 7.866*_3,63_**** | 5.05*** | 0.00006 | 0.0004*** | -0.000000004* |
| AFTrilm22 | log *f*(*SR*) = *β_0_ + β_1_*log*A* + *β_3_*(*logA*)(*logSE*) | 0.280 | 13.71*_2,64_**** | 1.6891*** | -0.2254*** | - | 0.0297*** |
| AFTrilm23 | *f*(*SR*) = *β_0_ + β_1_*log*A* + *β_3_*(*logA*)(*logSE*) | 0.310 | 16.11*_2,64_**** | 5.6679*** | -1.3772*** | - | 0.1957*** |
| AFTrilm24 | log *f*(*SR*) = *β_0_ + β_1_*log*A* + *β_3_*(*logA*)(*SE*) | 0.140 | 6.402*_2,64_*** | 1.6180*** | -0.0016 | - | 0.000003** |
| AFTrilm25 | *f*(*SR*) = *β_0_ + β_1_*log*A* + *β_3_*(*logA*)(*SE*) | 0.170 | 7.534*_2,64_*** | 5.1440*** | 0.1119 | - | 0.00002** |
| AFTrilm26 | log *f*(*SR*) = *β_0_ + β_1_A + β_3_*(*A*)(*logSE*) | 0.070 | 3.457*_2,64_** | 1.6870*** | -0.00004 | - | 0.00001 |
| AFTrilm27 | *f*(*SR*) = *β_0_ + β_1_A + β_3_*(*A*)(*logSE*) | 0.080 | 3.845*_2,64_** | 6.2640*** | -0.0002 | - | 0.00003 |
| AFTrilm28 | *f*(*SR*) = *β_0_ + β_1_A + β_3_*(*A*)(*SE*) | 0.053 | 2.836 | 6.103*** | 0.00005 | - | 0.0000000005 |

P-value significance is identified as follows ‘***’ 0.001 ‘**’ 0.01 ‘*’ 0.05.
