# Supplementary material for: Predictive sampling effort and species-area relationship models for estimating richness in fragmented landscapes
Source: PLoS One. 2019 Dec 31;14(12):e0226529. doi: 10.1371/journal.pone.0226529 (PMC6938349; doi:10.1371/journal.pone.0226529)
Supplement: S4 Table — (DOCX) [file pone.0226529.s005.docx]

**S4 Table. Results of multiple regression analyses for predicting species richness *f*(*SR*) for the endemic/forest specialist species of non-volant small mammals in Atlantic Forest remnants using 18 models that included both area of the forest remnants (*A*) and sampling effort (*S*) of the field studies.**

| **Model Name** | **Model** | **Adj R^2^** | **F Stat** | **β_0_ (Intercept)** | **β_1_ (Area)** | **β_2_ (Sampling)** | **β_3_ (Area:Sampling)** |
| --- | --- | --- | --- | --- | --- | --- | --- |
| AFTrilm1 | *f*(*SR*) = *β_0_* + *β_1_A + β_2_SE* | 0.022 | 1.727*_2,64_* | 3.267*** | 0.000009 | 0.00006 | - |
| AFTrilm2 | log *f*(*SR*) = *β_0_ + β_1_*log*A* + *β_2_*log*SE* | 0.029 | 1.975*_2,64_* | -0.01 | 0.0391 | 0.0997 | - |
| AFTrilm3 | *f*(*SR*) = *β_0_* + *β_1_*log*A* + *β_2_SE* | 0.081 | 3.894*_2,64_** | -0.79 | 0.0977 | 0.5007* | - |
| AFTrilm4 | log *f*(*SR*) = *β_0_ + β_1_*log*A* + *β_2_SE* | 0.024 | 1.82*_2,64_* | 0.66* | 0.0397 | 0.00001 | - |
| AFTrilm5 | *f*(*SR*) = *β_0_+ β_1_*log*A* + *β_2_SE* | 0.040 | 2.374*_2,64_* | 2.52** | 0.1354 | 0.00004 | - |
| AFTrilm6 | log *f*(*SR)* = *β_0_ + β_1_A* + *β_2_*log*SE* | 0.017 | 1.559*_2,64_* | 0.11 | 0.000003 | 0.1138 | - |
| AFTrilm7 | *f*(*SR*) = *β_0_* + *β_1_A + β_2_*log*SE* | 0.069 | 3.46*_2,64_** | -0.75 | 0.000002 | 0.5771* | - |
| AFTrilm8 | log *f(SR) =* *β_0_ + β_3_*(log*A*)(log*SE*) | 0.050 | 4.445*_1,65_** | 0.6016** | - | - | 0.0077* |
| AFTrilm9 | *f(SR) =* *β_0_ + β_3_*(log*A*)(log*SE*) | 0.010 | 1.693*_1,65_* | 0.9277*** | - | - | 0.0000007 |
| AFTrilm10 | log *f(SR) =* *β_0_ + β_3_*(*A*)(log*SE*) | 0.023 | 2.538*_1,65_* | 0.9057*** | - | - | 0.000002 |
| AFTrilm11 | *f(SR) =* *β_0_ + β_3_*(*A*)(log*SE*) | 0.079 | 6.629*_1,65_* | 2.2694*** | - | - | 0.02663* |
| AFTrilm12 | log *f(SR) =* *β_0_ + β_3_*(log*A*)(*SE*) | 0.018 | 2.201*_1,65_* | 3.41*** | - | - | 0.000002 |
| AFTrilm13 | *f(SR) =* *β_0_ + β_3_*(log*A*)(*SE*) | 0.030 | 3.047*_1,65_* | 3.349*** | - | - | 0.000006 |
| AFTrilm14 | *f(SR) =* *β_0_ + β_3_*(*A*)(*SE*) | -0.007 | 0.535*_1,65_* | 3.539*** | - | - | 0.0000000005 |
| AFTrilm16 | *f*(*SR*) = *β_0_ + β_1_*log*A* + *β_2_*log*SE* + *β_3_*(*logA*)(*logSE*) | 0.076 | 2.807*_3,63_** | 2.65 | -0.3584 | 0.0280 | 0.0609 |
| AFTrilm17 | log *f*(*SR*) = *β_0_ + β_1_*log*A* *+ β_2_SE + β_3_*(*logA*)(*SE*) | 0.014 | 1.305*_3,63_* | 0.56 | 0.0506 | 0.00006 | -0.000005 |
| AFTrilm18 | *f*(*SR*) = *β_0_ + β_1_*log*A + β_2_SE* + *β_3_*_(_*logA*)(*SE*) | 0.038 | 1.859*_3,63_* | 2.04* | 0.1861 | 0.0003 | -0.00002 |
| AFTrilm19 | log *f*(*SR*) = *β_0_ + β_1_A* + *β_2_*log*SE* + *β_3_*(*A*)(*logSE*) | 0.005 | 1.109*_3,63_* | -0.04 | 0.00003 | 0.1322 | -0.000003 |
| AFTrilm20 | *f*(*SR*) = *β_0_ + β_1_A + β_2_*log*SE* + *β_3_*(*A*)(*logSE*) | 0.068 | 0.068*_3,63_* | -1.56 | 0.0001 | 0.6767* | -0.00001 |
| AFTrilm21 | *f*(*SR*) = *β_0_ + β_1_A + β_2_SE* + *β_3_*(*A*)(*SE*) | 0.075 | 2.771*_3,63_* | 2.926*** | 0.00005 | 0.0001* | -0.000000003* |
| AFTrilm15 | log *f*(*SR*) = *β_0_ + β_1_*log*A + β_2_*log*SE + β_3_*(*logA*)(*logSE*) | 0.041 | 1.945*_3,63_* | 1.99 | -0.2272 | -0.1762 | 0.0356 |
| AFTrilm22 | log *f*(*SR*) = *β_0_ + β_1_*log*A* + *β_3_*(*logA*)(*logSE*) | 0.047 | 0.047*_2,64_** | 0.74** | -0.0895 | - | 0.0165 |
| AFTrilm23 | *f*(*SR*) = *β_0_ + β_1_*log*A* + *β_3_*(*logA*)(*logSE*) | 0.090 | 4.276*_2,64_** | 2.85*** | -0.3803 | - | 0.06398* |
| AFTrilm24 | log *f*(*SR*) = *β_0_ + β_1_*log*A* + *β_3_*(*logA*)(*SE*) | 0.021 | 1.716*_2,64_* | 0.68* | 0.0391 | - | 0.000001 |
| AFTrilm25 | *f*(*SR*) = *β_0_ + β_1_*log*A* + *β_3_*(*logA*)(*SE*) | 0.035 | 2.2*_2,64_* | 2.57** | 0.1367 | - | 0.000004 |
| AFTrilm26 | log *f*(*SR*) = *β_0_ + β_1_A + β_3_*(*A*)(*logSE*) | -0.005 | 0.852*_2,64_* | 0.92*** | 0.000009 | - | -0.0000002 |
| AFTrilm27 | *f*(*SR*) = *β_0_ + β_1_A + β_3_*(*A*)(*logSE*) | 0.004 | 1.13*_2,64_* | 3.38*** | 0.00004 | - | -0.000002 |
| AFTrilm28 | *f*(*SR*) = *β_0_ + β_1_A + β_3_*(*A*)(*SE*) | 0.020 | 1.669*_2,65_* | 3.3270 | 0.00005 | - | -0.000000001 |

P-value significance is identified as follows ‘***’ 0.001 ‘**’ 0.01 ‘*’ 0.05.
