# Supplementary material for: Predictive sampling effort and species-area relationship models for estimating richness in fragmented landscapes
Source: PLoS One. 2019 Dec 31;14(12):e0226529. doi: 10.1371/journal.pone.0226529 (PMC6938349; doi:10.1371/journal.pone.0226529)
Supplement: S5 Table — The predictors are smoothers for forest remnant area (A) and sampling effort (SE), with estimated degrees of freedom (e.d.f). (DOCX) [file pone.0226529.s006.docx]

**S5 Table Generalized additive model (GAM) results for species richness (*SR*) of the entire assemblage of non-volant small mammals in the Atlantic Forest. The predictors are smoothers for forest remnant area (*A*) and sampling effort (*SE*), with estimated degrees of freedom (e.d.f).**

| **Model name** | **Model** | **Coefficient** | **Std Error** | **t(F)-value** | **P-value** | **Adj. square** | **e.d.f** |
| --- | --- | --- | --- | --- | --- | --- | --- |
| AFGAM1 | log *f*(*SR*) = *y_i =_* *f_1_* + *f_2_*(log*A*) + *f_3_*(log*SE*) | *f_1_* (Intercept) | 0.05454 | 33.44 | < 0.001 | 0.393 |  |
|  |  | *f_2_* (log Area) |  | 0.405 | 0.527 |  | 1.00 |
|  |  | *f_3_* (log Sampling) |  | 39.501 | < 0.001 |  | 1.00 |
| AFGAM2 | *f*(*SR*) = *y_i =_* *f_1_* + *f_2_*(log*A*) + *f_3_*(log*SE*) | *f_1_* (Intercept) | 0.3695 | 19.65 | < 0.001 | 0.492 |  |
|  |  | *f_2_* (log A) |  | 0.177 | 0.676 |  | 1.00 |
|  |  | *f_3_* (log Sampling) |  | 15.786 | < 0.001 |  | 2.65 |
| AFGAM3 | log *f*(*SR*) = *y_i_* _=_ *f_1_* + *f_2_*(log*A*) + *f_3_*(*SE*) | *f_1_* (Intercept) | 0.05238 | 34.82 | < 0.001 | 0.440 |  |
|  |  | *f_2_* (log A) |  | 0.475 | 0.493 |  | 1.00 |
|  |  | *f_3_* (Sampling) |  | 15.813 | < 0.001 |  | 2.48 |
| AFGAM4 | *f*(*SR*) = *y_i_* _=_ *f_1_* + *f_2_*(log*A*) + *f_3_*(*SE*) | *f_1_* (Intercept) | 0.3648 | 19.91 | < 0.001 | 0.505 |  |
|  |  | *f_2_* (log A) |  | 0.073 | 0.789 |  | 1.00 |
|  |  | *f_3_* (Sampling) |  | 17.225 | < 0.001 |  | 2.62 |
| AFGAM5 | log *f*(*SR*) = *y_i =_* *f_1_* + *f_2_*(*A*) + *f_3_*(log*SE*) | *f_1_* (Intercept) | 0.05295 | 34.45 | < 0.001 | 0.428 |  |
|  |  | *f_2_* (A) |  | 0.0000 | 0.617 |  | 1.00 |
|  |  | *f_3_* (log Sampling) |  | 14.3620 | < 0.001 |  | 2.07 |
| AFGAM6 | *f*(*SR*) = *y_i_* _=_ *f_1_* + *f_2_*(*A*) + *f_3_*(log*SE*) | *f_1_* (Intercept) | 0.3708 | 19.58 | < 0.001 | 0.488 |  |
|  |  | *f_2_* (A) |  | 0.041 | 0.840 |  | 1.00 |
|  |  | *f_3_* (log Sampling) |  | 13.844 | < 0.001 |  | 2.57 |
