# Supplementary material for: Predictive sampling effort and species-area relationship models for estimating richness in fragmented landscapes
Source: PLoS One. 2019 Dec 31;14(12):e0226529. doi: 10.1371/journal.pone.0226529 (PMC6938349; doi:10.1371/journal.pone.0226529)
Supplement: S6 Table — The predictors are smoothers for forest remnant area (A) and sampling effort (S), with estimated degrees of freedom (e.d.f.). (DOCX) [file pone.0226529.s007.docx]

**S6 Table. Generalized additive model (GAM) results for species richness (*SR*) of the native species forest assemblage of non-volant small mammal assemblage in the Atlantic Forest. The predictors are smoothers for forest remnant area (*A*) and sampling effort (*S*), with estimated degrees of freedom (e.d.f.).**

| **Model name** | **Model** | **Coefficient** | **Std Error** | **t(F)-value** | **P-value** | **Adj. square** | **e.d.f** |
| --- | --- | --- | --- | --- | --- | --- | --- |
| AFGAM1_For | log *f*(*SR*) = *y_i =_* *f_1_* + *f_2_*(log*A*) + *f_3_*(log*SE*) | *f_1_* (Intercept) | 0.058 | 29.54 | < 0.001 | 0.332 |  |
|  |  | *f_2_* (log Area) |  | 0.275 | 0.602 |  | 1.00 |
|  |  | *f_3_* (log Sampling) |  | 30.600 | < 0.001 |  | 1.00 |
| AFGAM2_For | *f*(*SR*) = *y_i =_* *f_1_* + *f_2_*(log*A*) + *f_3_*(log*SE*) | *f_1_* (Intercept) | 0.365 | 18.040 | < 0.001 | 0.407 |  |
|  |  | *f_2_* (log Area) |  | 0.403 | 0.52800 |  | 1.00 |
|  |  | *f_3_* (log Sampling) |  | 9.663 | < 0.001 |  | 3.05 |
| AFGAM3_For | log *f*(*SR*) = *y_i =_* *f_1_* + *f_2_*(log*A*) + *f_3_*(*SE*) | *f_1_* (Intercept) | 0.057 | 30.45 | < 0.001 | 0.371 |  |
|  |  | *f_2_* (log Area) |  | 0.068 | 0.795 |  | 1.00 |
|  |  | *f_3_* (Sampling) |  | 11.499 | < 0.001 |  | 2.56 |
| AFGAM4_For | *f*(*SR*) = *y_i =_* *f_1_* + *f_2_*(log*A*) + *f_3_*(*SE*) | *f_1_* (Intercept) | 0.352 | 18.720 | < 0.001 | 0.449 |  |
|  |  | *f_2_* (log Area) |  | 0.305 | 0.583 |  | 1.00 |
|  |  | *f_3_* (Sampling) |  | 12.895 | < 0.001 |  | 2.85 |
| AFGAM5_For | log *f*(*SR*) = *y_i =_* *f_1_* + *f_2_*(*A*) + *f_3_*(log*SE*) | *f_1_* (Intercept) | 0.058 | 29.640 | < 0.001 | 0.337 |  |
|  |  | *f_2_* (Area) |  | 0.706 | 0.404 |  | 1.00 |
|  |  | *f_3_* (log Sampling) |  | 29.105 | < 0.001 |  | 1.00 |
| AFGAM6_For | *f*(*SR*) = *y_i =_* *f_1_* + *f_2_*(*A*) + *f_3_*(log*SE*) | *f_1_* (Intercept) | 0.366 | 18.00 | < 0.001 | 0.405 |  |
|  |  | *f_2_* (Area) |  | 1.307 | 0.608 |  | 1.31 |
|  |  | *f_3_* (log Sampling) |  | 2.646 | < 0.001 |  | 2.65 |
