# Supplementary material for: Predictive sampling effort and species-area relationship models for estimating richness in fragmented landscapes
Source: PLoS One. 2019 Dec 31;14(12):e0226529. doi: 10.1371/journal.pone.0226529 (PMC6938349; doi:10.1371/journal.pone.0226529)
Supplement: S7 Table — The predictors are smoothers for forest remnant area (A) and sampling effort (S), with estimated degrees of freedom (e.d.f.). (DOCX) [file pone.0226529.s008.docx]

**S7 Table Generalized additive model (GAM) results for species richness (*SR*) of the forest-specialist assemblage of non-volant small mammals in the Atlantic Forest. The predictors are smoothers for forest remnant area (*A*) and sampling effort (*S*), with estimated degrees of freedom (e.d.f.).**

| **Model name** | **Model** | **Coefficient** | **Std Error** | **t(F)-value** | **P-value** | **Adj. square** | **e.d.f** |
| --- | --- | --- | --- | --- | --- | --- | --- |
| AFGAM1_End | Log *f*(*SR*) = y_i =_ *f_1_* + *f_2_*(log*A*) + *f_3_*(log*SE*) | *f_1_* (Intercept) | 0.105 | 9.449 | < 0.001 | 0.029 |  |
|  |  | *f_2_* (log Area) |  | 1.007 | 0.319 |  | 1.00 |
|  |  | *f_3_* (log Sampling) |  | 1.249 | 0.268 |  | 1.00 |
| AFGAM2_End | *f*(*SR*) = *y_i_* _=_ *f_1_* + *f_2_*(log*A*) + *f_3_*(log*SE*) | *f_1_* (Intercept) | 0.278 | 12.980 | < 0.001 | 0.172 |  |
|  |  | *f_2_* (log Area) |  | 1.397 | 0.242 |  | 1.00 |
|  |  | *f_3_* (log Sampling) |  | 2.523 | 0.0376 |  | 3.02 |
| AFGAM3_End | log *f*(*SR*) = *y_i =_* *f_1_* + *f_2_*(log*A*) + *f_3_*(*SE*) | *f_1_* (Intercept) | 0.103 | 9.554 | < 0.001 | 0.050 |  |
|  |  | *f_2_* (log Area) |  | 0.948 | 0.334 |  | 1.00 |
|  |  | *f_3_* (Sampling) |  | 0.582 | 0.361 |  | 1.33 |
| AFGAM4_End | *f*(*SR*) = *y_i =_* *f_1_* + *f_2_*(log*A*) + *f_3_*(*SE*) | *f_1_* (Intercept) | 0.277 | 13.01 | < 0.001 | 0.176 |  |
|  |  | *f_2_* (log Area) |  | 1.219 | 0.274 |  | 1.00 |
|  |  | *f_3_* (Sampling) |  | 4.268 | 0.013 |  | 2.19 |
| AFGAM5_End | log *f*(*SR*) = *y_i =_* *f_1_* + *f_2_*(*A*) + *f_3_*(log*SE*) | *f_1_* (Intercept) | 0.105 | 9.391 | < 0.001 | 0.017 |  |
|  |  | *f_2_* (Area) |  | 0.212 | 0.647 |  | 1.00 |
|  |  | *f_3_* (log Sampling) |  | 1.380 | 0.244 |  | 1.00 |
| AFGAM6_End | *f*(*SR*) = *y_i_* _=_ *f_1_* + *f_2_*(*A*) + *f_3_*(log*SE*) | *f_1_* (Intercept) | 0.270 | 13.370 | < 0.001 | 0.220 |  |
|  |  | *f_2_* (Area) |  | 1.819 | 0.12100 |  | 2.08 |
|  |  | *f_3_* (log Sampling) |  | 1.974 | 0.0832 |  | 2.77 |
