# Supplementary material for: Predictive sampling effort and species-area relationship models for estimating richness in fragmented landscapes
Source: PLoS One. 2019 Dec 31;14(12):e0226529. doi: 10.1371/journal.pone.0226529 (PMC6938349; doi:10.1371/journal.pone.0226529)
Supplement: S8 Table — The maximum forest remnant size was 184,553.80 ha. (DOCX) [file pone.0226529.s009.docx]

**S8 Table. Predicted species richness of non-volant small mammals in Paraguayan Atlantic Forest remnants varies based on size of the forest remnant and the species assemblage (entire, native species forest or forest-specialist). The maximum forest remnant size was 184,553.80 ha.**

|  | **Species Richness** | | | | | |
| --- | --- | --- | --- | --- | --- | --- |
| **Assemblage** | 2-3 | 4-5 | 6-7 | 8-9 | 10-12 |  |
| **Entire** |  |  |  |  |  |  |
| Max. remnant size (ha) | - | - | 350.39 | 56,152.95 | 184,553.80 |  |
| # of remnants | - | - | 140,231 | 678 | 4 |  |
| % of remnants | - | - | 99.52% | 0.48% | <0.01% |  |
| **Native Species Forest** |  |  |  |  |  |  |
| Max. remnant size (ha) | - | 77.06 | 2,522.43 | 184,553.80 | - |  |
| # of remnants | - | 138,024 | 2,793 | 96 | - |  |
| % of remnants | - | 97.95% | 1.98% | 0.07% | - |  |
| **Forest-specialist** |  |  |  |  |  |  |
| Max. remnant size (ha) | 15,844.62 | 184,553.80 | - | - | - |  |
| # of remnants | 140,898 | 15 | - | - | - |  |
| % of remnants | 99.99% | 0.01% | - | - | - |  |

^a^Data are based on modeling of all Atlantic Forest remnants 0.5 ha and larger in Paraguay.
